# Angiopoietin-like 8 governs osteoblast-adipocyte lineage commitment during skeletal aging

**DOI:** 10.1172/jci.insight.189371

**Published:** 2025-10-21

**Authors:** Yaming Guo, Zeqing Zhang, Junyu He, Peiqiong Luo, Zhihan Wang, Yurong Zhu, Xiaoyu Meng, Limeng Pan, Ranran Kan, Yuxi Xiang, Beibei Mao, Yi He, Siyi Wang, Yan Yang, Fengjing Guo, Hongbo You, Feng Li, Danpei Li, Yong Chen, Xuefeng Yu

**Affiliations:** 1 Division of Endocrinology, Department of Internal Medicine, Tongji Hospital, Tongji Medical College, Huazhong University of Science and Technology, Wuhan, China.; 2 Hubei Clinical Medical Research Center for Endocrinology and Metabolic Diseases, Hubei, China.; 3 Branch of National Clinical Research Center for Metabolic Diseases, Hubei, China.; 4 Department of Orthopedics, Tongji Hospital, Tongji Medical College, Huazhong University of Science and Technology, Wuhan, China.

**Keywords:** Aging, Endocrinology, Adult stem cells, Bone disease, Bone marrow differentiation

## Abstract

A distinguishing feature of older mesenchymal stem cells (MSCs) from bone marrow (BM) is the transition in their differentiation capabilities from osteoblasts to adipocytes. However, the mechanisms underlying these cellular events during the aging process remain unclear. We identified angiopoietin-like protein 8 (ANGPTL8), an adipokine implicated in lipid metabolism, that influenced the fate of MSCs in BM during skeletal aging. Our studies revealed that ANGPTL8 steered MSCs toward adipogenic differentiation, overshadowing osteoblastogenesis. Mice with overexpressed ANGPTL8 exhibited reduced bone mass and increased BM adiposity, while those with transgenic depletion of ANGPTL8 showed lowered bone loss and less accumulation of BM fat. ANGPTL8 influenced the BM niche of MSCs by inhibiting the Wnt/β-catenin signaling pathway. Partial inhibition of PPARγ rescued some aspects of the phenotype in MSCs with ANGPTL8 overexpression. Furthermore, treatment with an *Angptl8* antisense oligonucleotide improved the phenotype of aging mice. Our research suggests that ANGPTL8 is a crucial regulator of senesence-related changes in the BM niche and the cell-fate switch of MSCs.

## Abstract 2

ANGPTL8 is a critical regulator of senesence-related changes in the bone marrow niche and the cell fate switch of mesenchymal stem cells.

## Graphical abstract

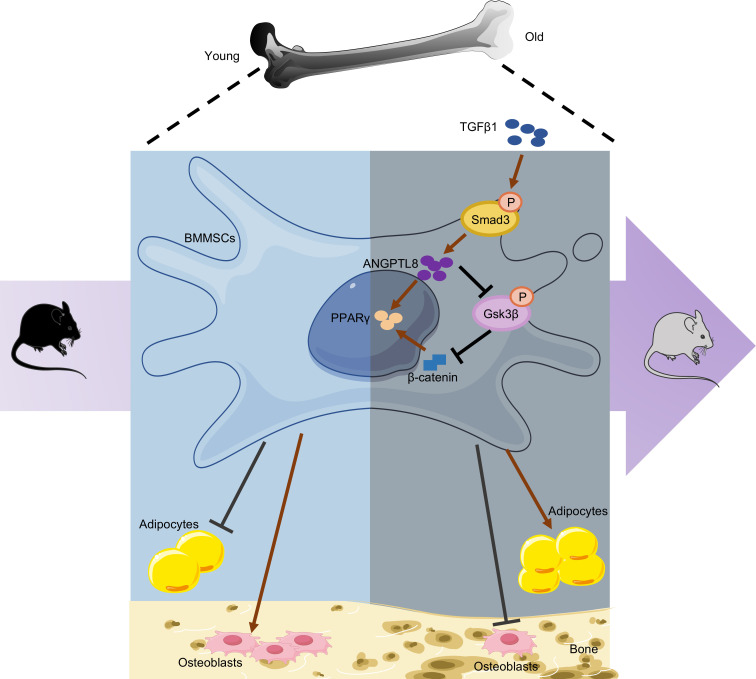

## Introduction

Guided by genetic and molecular regulators, along with the surrounding microenvironment, mesenchymal stem cells (MSCs) in bone marrow (BM) go through differentiation processes leading to the development of different mature cells, such as adipocytes and osteoblasts (1–3). Anatomically, MSCs are characterized as PDGF-α^+^Sca1^+^CD31^–^CD45^–^ and are predominantly located within the perivasculature, a site that can be characteristically targeted using Nestin-Cre (4, 5), Prx1-Cre (6), or Lepr-Cre (7). With age, MSCs tend to differentiate more into adipocytes than into osteoblasts, leading to an increase in adipocytes and a decrease in osteoblasts, ultimately triggering osteoporosis (8–10); however, the molecular mechanisms behind this shift are not fully understood.

Angiopoietin-like protein 8 (ANGPTL8), also referred to as betatrophin, is an adipokine with essential functions in inflammation and is associated with metabolic and aging-related disorders such as obesity, diabetes (11), hypothyroidism, nonalcoholic steatohepatitis (12), and polycystic ovary syndrome (13, 14). ANGPTL8 is primarily produced in the liver and, to a lesser extent, in adipose tissues (15), promoting adipogenesis in adipose stem cells (16). Yet, limited studies have been conducted on the impact of ANGPTL8 on the change in lineage fate of MSCs related to aging within the skeletal system.

Our current research revealed ANGPTL8 as a highly reactive element in BM adipose tissue (BMAT) expansion in both mice and humans. We utilized various models with ANGPTL8 overexpression or knockout to systematically investigate the function and regulation of ANGPTL8 in MSCs in BM. Our results indicate that ANGPTL8 could be a promising treatment target for age-related osteoporosis.

## Results

### ANGPTL8 expression increased with age in BM during skeletal aging.

**Figure 1:**
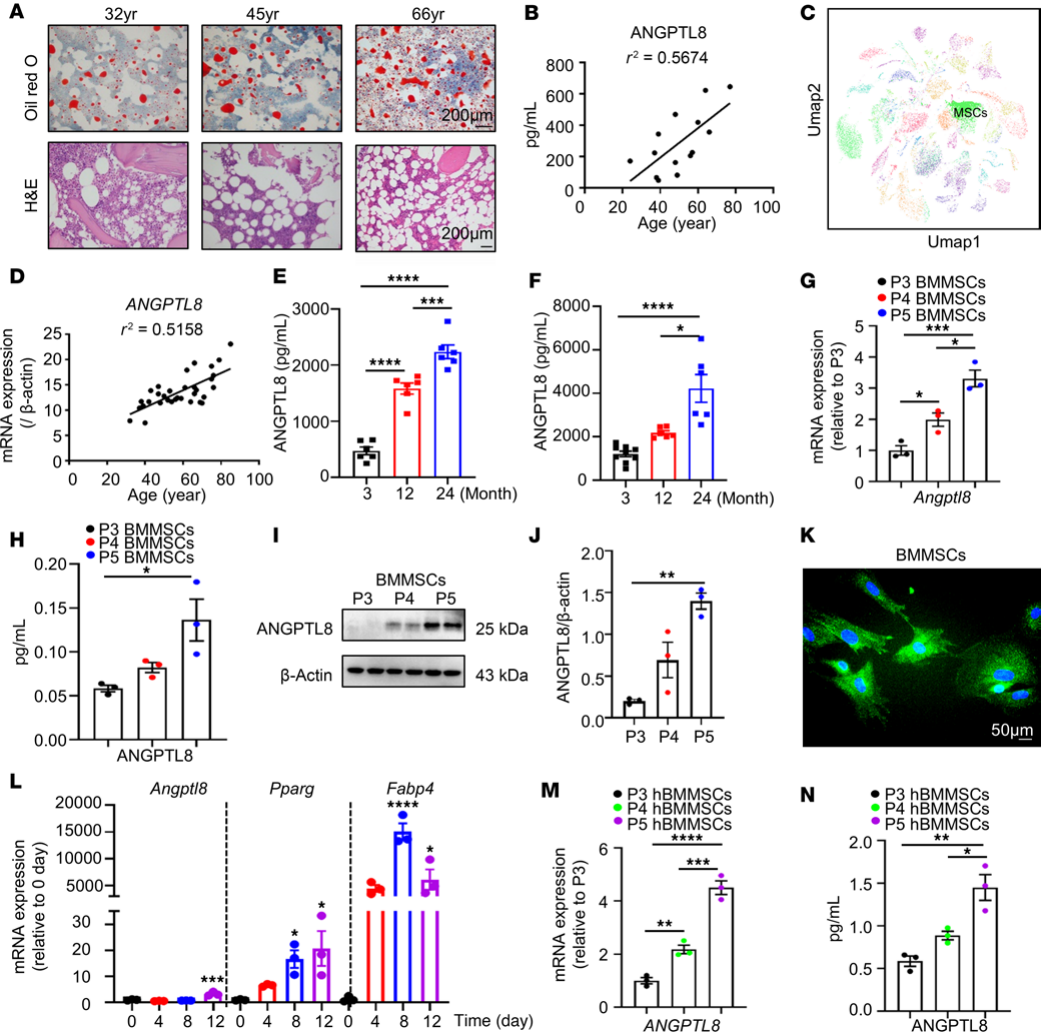
ANGPTL8 expression increases with age in BM during skeletal aging. (**A**) Oil Red O and H&E staining of human BM samples from individuals at different ages. Scale bars: 200 μm. (**B**) ANGPTL8 levels in BM supernatants (*n* = 14) from different individuals. (**C**) Bioinformatics analysis of single-cell RNA sequencing of BM cells expressing ANGPTL8 from old (24 months) WT male mice. (**D**) Age-associated *ANGPTL8* mRNA expression in hBMMSCs (*n* = 33). (**E** and **F**) ANGPTL8 levels in BM supernatants (**E**) and serum (**F**) from 3-, 12-, and 24-month-old WT male mice (*n* = 6). (**G**–**J**) mRNA expression of *Angptl8* (**G**), corresponding ANGPTL8 levels in supernatants (**H**), and Western blot analysis for ANGPTL8 (**I** and **J**) in 18-month-old male mice BMMSCs from different cell passages (*n* = 3). (**K**) Representative images showing immunofluorescent staining of ANGPTL8 (green) in passage 5 (P5) BMMSCs from 18-month-old male mice. Scale bar: 50 μm. (**L**) qRT-PCR analysis of the mRNA expression of *Angptl8*, *Pparg*, and *Fabp4* during the differentiation of BMMSCs from 3-month-old WT male mice into adipocytes (*n* = 3). (**M** and **N**) mRNA expression of *ANGPTL8* (**M**) and ANGPTL8 levels (**N**) in supernatants from hBMMSCs from different passages (*n* = 3). Data are mean ± SEM. *P < 0.05; **P < 0.01; ***P < 0.001; ****P < 0.0001 by 1-way ANOVA followed by Tukey’s multiple-comparison test.

To identify dysregulated ANGPTL8 in MSCs from BM during skeletal aging, we collected BM samples from individuals of different ages and observed that BMAT accumulation increased with age (Figure 1A), as indicated by H&E and Oil Red O staining. Meanwhile, ANGPTL8 levels progressively rose in human BM supernatants (Figure 1B). Based on a single-cell sequencing database (17), we observed expression of ANGPTL8 in MSCs derived from BM cells of 24-month-old male mice (Figure 1C). To determine whether this age-related expression is conserved in humans, we examined ANGPTL8 levels in human BM MSCs (hBMMSCs) from donors of different ages. Consistent with the murine data, *ANGPTL8* expression in hBMMSCs also increased with age (Figure 1D). Moreover, adipogenic markers (*PPARG*, *CEBPβ*, and *FABP4*) and cellular senescence markers (*P16* and *P21*) in hBMMSCs positively correlated with age, whereas osteogenic markers (*ALP*, *RUNX2*, and *SP7*) were inversely correlated with age (Supplemental Figure 1, A–H; supplemental material available online with this article; https://doi.org/10.1172/jci.insight.189371DS1). Interestingly, the relationships between these markers and *ANGPTL8* were similar to that with age (Supplemental Figure 1, I–P).

In vivo studies in 3-, 12-, and 24-month-old wild-type (WT) mice revealed that ANGPTL8 in BM supernatants (Figure 1E) and serum (Figure 1F) also increased with age. We sorted mouse BMMSCs, defined as STRO-1^+^CD146^+^CD45^–^CD11b^–^ (Supplemental Figure 2A) by flow cytometry, and observed that ANGPTL8 concurrently increased from passage 3 to passage 5 (P3 to P5; Figure 1, G–J). Cellular immunofluorescence localization demonstrated that ANGPTL8 was expressed in BMMSCs (Figure 1K). During the differentiation of BMMSCs to adipocytes, the relative expression of *Angptl8* increased with time (Figure 1L). Notably, these findings were replicated in cultures of hBMMSCs expanded in vitro for 3 passages (P3 to P5; Figure 1, M–N).

During the aging process, the BM microenvironment experiences age-related changes. Among these are changes in various bone-related hormones (e.g., estrogen) (18), growth factor signaling (e.g., TGF-β) (19–21), and age-associated stress (e.g., oxidative stress) (22, 23). To further explore what regulates ANGPTL8 in BMMSCs during aging, we first tested the impact of estrogen and buthionine-sulfoximine (BSO, a potent inhibitor of glutamylcysteine synthetase biosynthesis) (24) on ANGPTL8 in MSCs. Somewhat unexpectedly, neither of these factors influenced *Angptl8* expression in BMMSCs and hBMMSCs (Supplemental Figure 2, B–E). Levels of TGF-β1, -β2, and -β3 increased with age in human BM samples (Supplemental Figure 2, F–H) and in mouse serum (Supplemental Figure 2, I–K). Given that TGF-β1 was the most abundant isoform among these TGF-β family members, we selected it for subsequent treatment of BMMSCs. Surprisingly, TGF-β1 upregulated *Angptl8* in a manner that was dependent on the dosage (Supplemental Figure 2, L and M). Previous studies have shown that upregulation of TGF-β1 in renal tubular epithelial cells following Bmi1 deletion activates the p53/Smad3 axis, thereby promoting epithelial-mesenchymal transition and cellular senescence (25). In our study, treatment of BMMSCs with TGF-β1 increased *Angptl8* expression; this effect was attenuated by transfection with siRNA against *Smad3* (siSmad3) (Supplemental Figure 2N). Together, these findings indicate that the TGF-β pathway promotes age-related upregulation of *Angptl8* in MSCs through Smad3 activation.

These observations imply that ANGPTL8 levels increase with age in BM during skeletal aging. Additionally, ANGPTL8 from BM is positively correlated with adipogenesis and cellular senescence, whereas it is negatively correlated with osteoblastogenesis.

### ANGPTL8 modulated cell-fate choice of MSCs between adipocytes and osteoblasts.

**Figure 2:**
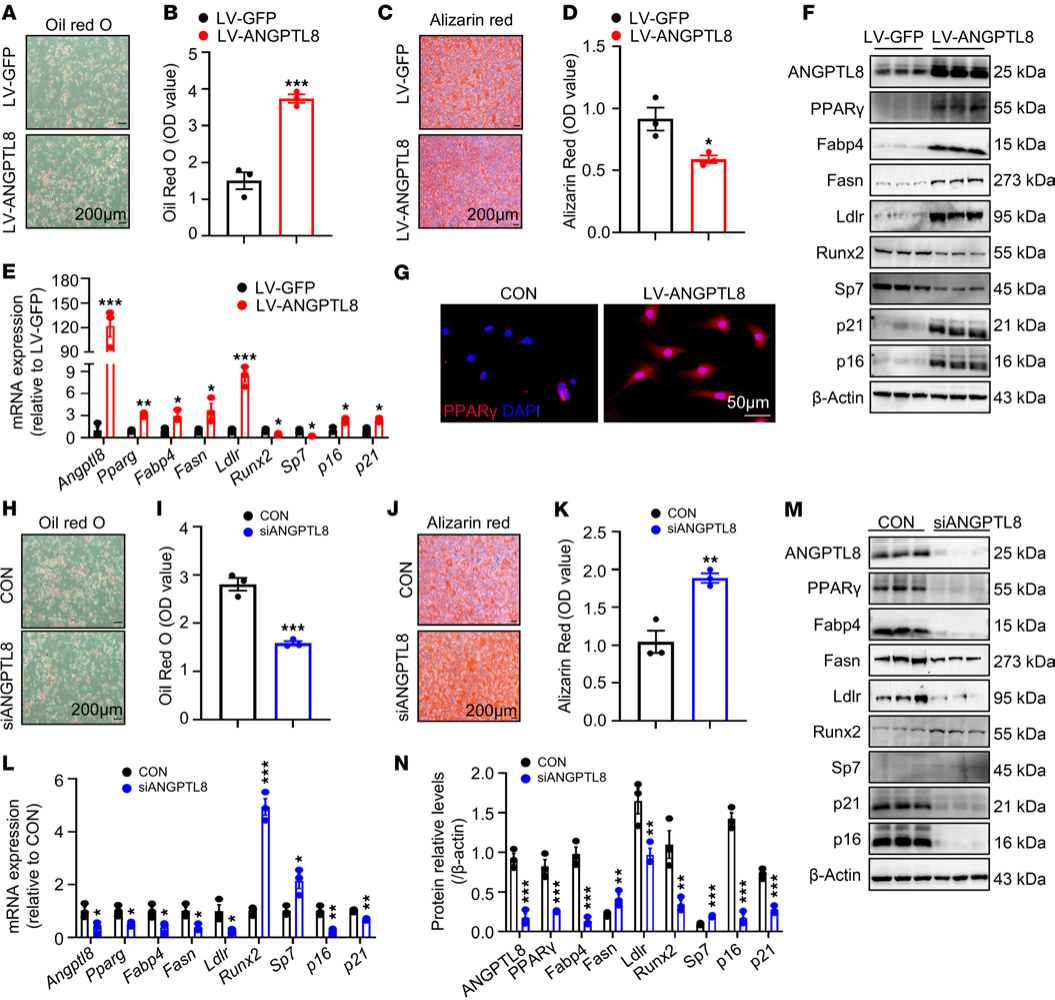
ANGPTL8 modulates cell-fate choice of MSCs between adipocytes and osteoblasts. (**A**) Cell differentiation was assessed 14 days after adipogenic induction by Oil Red O staining in BMMSCs transfected with LV-GFP or LV-ANGPTL8. Scale bar: 50 μm. (**B**) Quantification of Oil Red O staining in **A**. *n* = 3. (**C**) Cell differentiation was assessed 14 days after osteogenic induction by alizarin red staining in BMMSCs transfected with lentiviral LV-GFP or LV-ANGPTL8. Scale bar: 200 μm. (**D**) Quantification of calcium mineralization based on alizarin red staining in **C**. *n* = 3. (**E**) qRT-PCR analysis of the mRNA expression of *Angptl8*, *Pparg*, *Fabp4*, *Fasn*, *Ldlr*, *Runx2*, *Sp7*, *p16*, and *p21* in ANGPTL8-overexpressing BMMSCs. (**F**) Western blot analysis of ANGPTL8, PPARγ, Fabp4, Fasn, Ldlr, Runx2, Sp7, p16, and p21 in BMMSCs after transfection with LV-GFP or LV-ANGPTL8. (**G**) Representative images showing immunofluorescent staining of PPARγ in BMMSCs treated with ANGPTL8. Scale bar: 50 μm. (**H**) The adipogenic potential of BMMSCs after transfected with siANGPTL8 was assessed 14 days after induction of differentiation by Oil Red O staining. Scale bar: 50 μm. (**I**) Quantification of Oil Eed O staining in **H**. *n* = 3. (**J**) The osteogenic potential of BMMSCs after transfection with siANGPTL8 was assessed 14 days after induction of differentiation by alizarin red staining. Scale bar: 200 μm. (**K**) Quantification of calcium mineralization based on alizarin red staining in **J**. *n* = 3. (**L**) qRT-PCR analysis of the mRNA expression of *Angptl8*, *Pparg*, *Fabp4*, *Fasn*, *Ldlr*, *Runx2*, *Sp7*, *p16*, and *p21* in BMMSCs after transfection with siANGPTL8. (**M**) Western blot analysis of ANGPTL8, PPARγ, Fabp4, Fasn, Ldlr, Runx2, Sp7, p16, and p21 in BMMSCs after transfection with siANGPTL8. (**N**) ANGPTL8, PPARγ, Fabp4, Fasn, Ldlr, Runx2, Sp7, p16, and p21 expression relative to β-actin was assessed by densitometric analysis from BMMSCs transfected with siANGPTL8. *n* = 3. Data are mean ± SEM. *P < 0.05; **P < 0.01; ***P < 0.001 by 2-tailed Student’s *t* test.

To explore the role of ANGPTL8 in cell-fate decision making, MSCs were sourced from BM of WT mice and transfected with lentivirus overexpressing ANGPTL8 (LV-ANGPTL8) or siRNA against *Angptl8* (siANGPTL8) to either overexpress or silence ANGPTL8, respectively. Over a period of 14 days after being induced toward adipogenic or osteogenic pathways, these cells more easily differentiated into adipocytes, as indicated by increased Oil Red O staining for lipid droplets (Figure 2, A and B). Conversely, the osteogenic capacity of BMMSCs transfected with LV-ANGPTL8 was reduced, as evidenced by decreased alizarin red staining for mineralization (Figure 2, C and D). The qRT-PCR data indicated a progressive increase in *Angptl8* expression in BMMSCs transfected with LV-ANGPTL8 (Figure 2E), accompanied by elevated mRNA levels of key adipogenic markers *Pparg*, *Fabp4*, *Fasn*, and *Ldlr* along with aging markers *p16* and *p21* (Figure 2E). Conversely, mRNA expression of osteoblast markers, including *Runx2* and *Sp7* (Figure 2E), decreased. Western blot analysis and immunofluorescent staining further confirmed these findings (Figure 2, F and G, and Supplemental Figure 3A). By contrast, ANGPTL8 silencing attenuated adipogenic differentiation in BMMSCs, as evidenced by reduced lipid droplet accumulation (Figure 2, H and I), and downregulated the mRNA expression of adipogenic markers (*Pparg*, *Fabp4*, *Fasn*, and *Ldlr*) and senescence-related genes (*p16* and *p21*) (Figure 2L). Conversely, it enhanced osteogenic differentiation (Figure 2, J and K) and elevated the expression of osteogenic markers *Runx2* and *Sp7* (Figure 2L). These findings were further confirmed at the protein level by Western blot analysis (Figure 2, M and N).

To confirm the role of ANGPTL8 in regulating the differentiation of hBMMSCs across the population, we transfected hBMMSCs with LV-ANGPTL8 and induced lipogenic and osteogenic differentiation. We reached similar conclusions in hBMMSCs (Supplemental Figure 4) and the cell line C3H10T1/2 (Supplemental Figure 5).

Collectively, these in vitro results suggest that ANGPTL8 directly regulated the fates of MSCs from BM by favoring their differentiation into adipocytes and impeding osteoblastogenesis.

### Angptl8-_Nestin-cre_Tg mice displayed reduced bone mass and higher BMAT deposition.

**Figure 3:**
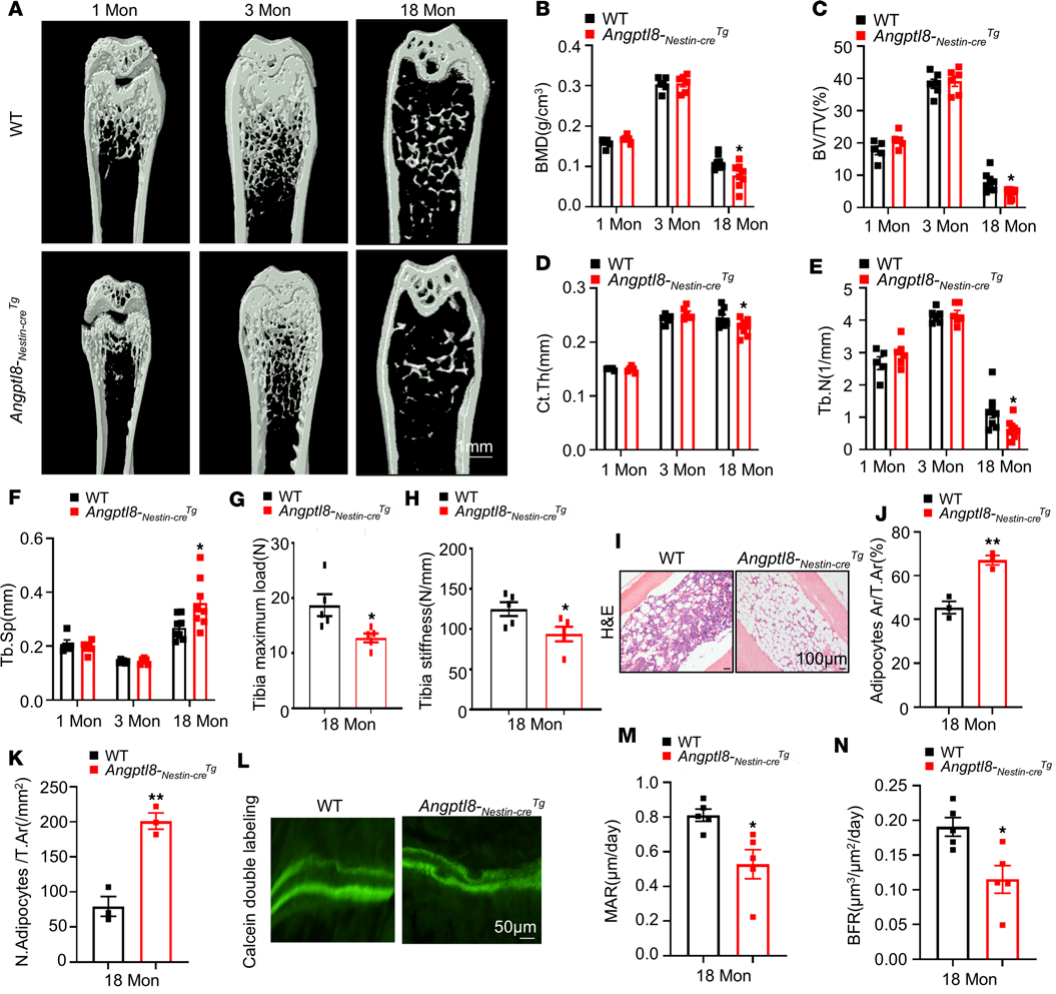
*Angptl8-_Nestin-cre_Tg* mice display lower bone mass and higher fat accumulation in BM. (**A**) Representative microCT images of femurs from 1-, 3-, and 18-month-old WT and *Angptl8-_Nestin-cre_Tg* male mice. Scale bar: 1 mm. (**B**–**F**) Quantitative microCT analysis of the femurs from 1-, 3-, and 18-month-old WT and *Angptl8-_Nestin-cre_Tg* male mice. *n* = 6–8. BMD, bone mineral density; BV/TV, bone volume per tissue volume; Ct.Th, cortical bone thickness; Tb.N, trabecular number; Tb.Sp, trabecular separation. (**G** and **H**) Three-point bending measurement of tibia maximum load (**G**) and stiffness (**H**) from 18-month-old WT and *Angptl8-_Nestin-cre_Tg* male mice. *n* = 5. (**I**) H&E staining for BMAT in the femurs of 18-month-old WT and *Angptl8-_Nestin-cre_Tg* male mice. Scale bars: 100 μm. (**J** and **K**) Quantification of area (**J**) and number of adipocytes (**K**) in the femurs of 18-month-old WT and *Angptl8-_Nestin-cre_Tg* male mice. *n* = 3. (**L**) Representative images of calcein double labeling of trabecular bone from 18-month-old WT and *Angptl8-_Nestin-cre_Tg* male mice. Scale bar: 50 μm. (**M** and **N**) Quantification of the mineral apposition rate (MAR) and bone formation rate (BFR) based on calcein double labeling from 18-month-old WT and *Angptl8-_Nestin-cre_Tg* male mice. *n* = 5. Data are mean ± SEM. *P < 0.05; **P < 0.01 by 2-tailed Student’s *t* test.

To assess the role of ANGPTL8 in MSC differentiation and senescence in vivo, we activated it specifically in BMMSCs by crossing *Angptl8^fl/fl^* with *Nestin*-Cre. We found that 18-month-old *Angptl8-_Nestin-cre_Tg* mice exhibited reduced bone volume/total volume (BV/TV), cortical bone thickness (Ct.th), bone mineral density (BMD), and trabecular bone volume number (Tb.N) but higher trabecular separation (Tb.Sp) in femurs relative to their WT littermates, as shown by microcomputed tomography (microCT) analysis (Figure 3, A–F). Correspondingly, 18-month-old *Angptl8-_Nestin-cre_Tg* mice exhibited decreased tibia maximum load and stiffness values, indicating compromised bone strength compared with WT counterparts (Figure 3, G and H). In aged, but not young, *Angptl8-_Nestin-cre_Tg* mice, a marked increase in both the number and area of BM adipocytes was observed (Figure 3, I–K). Furthermore, calcein double labeling demonstrated that the trabecular bone mineral apposition rate (MAR) and bone formation rate (BFR) were substantially lower in *Angptl8-_Nestin-cre_Tg* mice compared with their WT littermates (Figure 3, L–N). We also explored the role of ANGPTL8 in systemically ANGPTL8-overexpressing mice (*Angptl8-_Cagg-cre_Tg*), finding that *Angptl8-_Cagg-cre_Tg* mice exhibited reduced bone mass and higher BMAT accumulation (Supplemental Figure 6). Thus, we conclude that mice overexpressing *Angptl8* have reduced bone mass and increased BMAT deposition.

### Angptl8^–/–^ mice exhibited higher bone mass and reduced BM adiposity.

**Figure 4:**
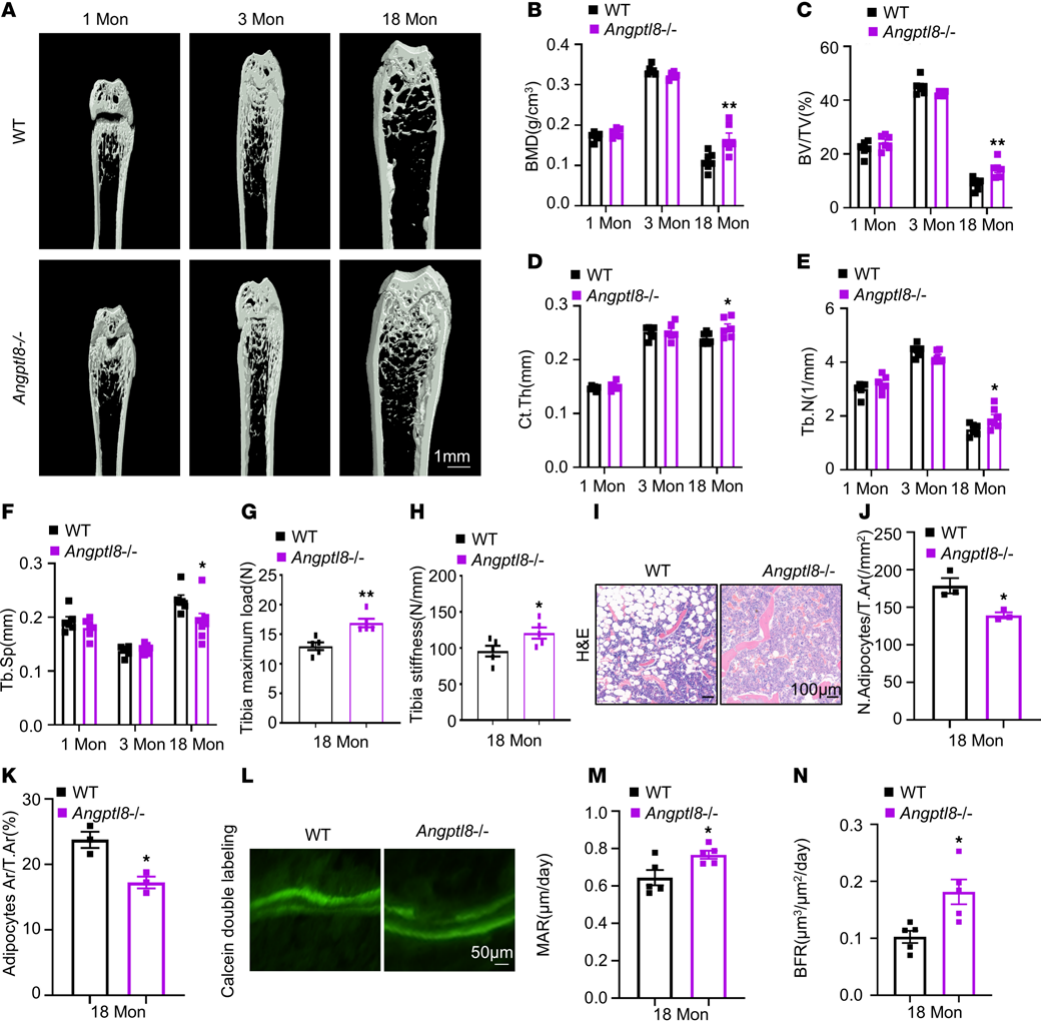
*Angptl8*^–/–^ mice exhibit higher bone mass and lower BMAT accumulation. (**A**) Representative microCT images of femurs from 1-, 3-, and 18-month-old WT and *Angptl8^–/–^* male mice. Scale bar: 1 mm. (**B**–**F**) Quantitative microCT analysis of the femurs from 1-, 3-, and 18-month-old WT and *Angptl8^–/–^* male mice. *n* = 6–8. BMD, bone mineral density; BV/TV, bone volume per tissue volume; Ct.Th, cortical bone thickness; Tb.N, trabecular number; Tb.Sp, trabecular separation. (**G** and **H**) Three-point bending measurement of tibia maximum load (**G**) and stiffness (**H**) from 18-month-old WT and *Angptl8^–/–^* male mice. *n* = 5. (**I**) H&E staining for BMAT in the femurs of 18-month-old WT and *Angptl8^–/–^* male mice. Scale bars: 100 μm. (**J** and **K**) Quantification of number (**J**) and area of adipocytes (**K**) in the femurs of 18-month-old WT and *Angptl8^–/–^* male mice. *n* = 3. (**L**) Representative images of calcein double labeling of trabecular bone from 18-month-old WT and *Angptl8^–/–^* male mice. Scale bar: 50 μm. (**M** and **N**) Quantification of the mineral apposition rate (MAR) and bone formation rate (BFR) based on calcein double labeling from 18-month-old WT and *Angptl8^–/–^* male mice. *n* = 5. Data are mean ± SEM. *P < 0.05; **P < 0.01 by 2-tailed Student’s *t* test.

We constructed knockout mice with silenced ANGPTL8 to explore whether ablation of ANGPTL8 in vivo would mitigate bone loss and BMAT accumulation. Strikingly, microCT analysis revealed that the femurs of 18-month-old *Angptl8*^–/–^ male mice exhibited substantial bone changes compared with their WT littermates. Specifically, BV/TV, Ct.Th, BMD, and Tb.N were increased, while Tb.Sp was decreased (Figure 4, A–F). Additionally, values of tibia maximum load and stiffness, representing bone strength, were higher in aged *Angptl8*^–/–^ mice than in WT controls (Figure 4, G and H). The number and area of adipocytes were reduced in 18-month-old *Angptl8*^–/–^ male mice compared with WT controls (Figure 4, I–K). Furthermore, calcein double labeling showed that *Angptl8*^–/–^ mice exhibited substantially higher trabecular MAR and BFR relative to their WT littermates (Figure 4, L–N). These findings reinforce our hypothesis that BM-derived ANGPTL8 favors BMAT development while negatively impacting bone well-being.

### ANGPTL8 regulated MSC differentiation by Wnt/β-catenin signaling pathway.

**Figure 5:**
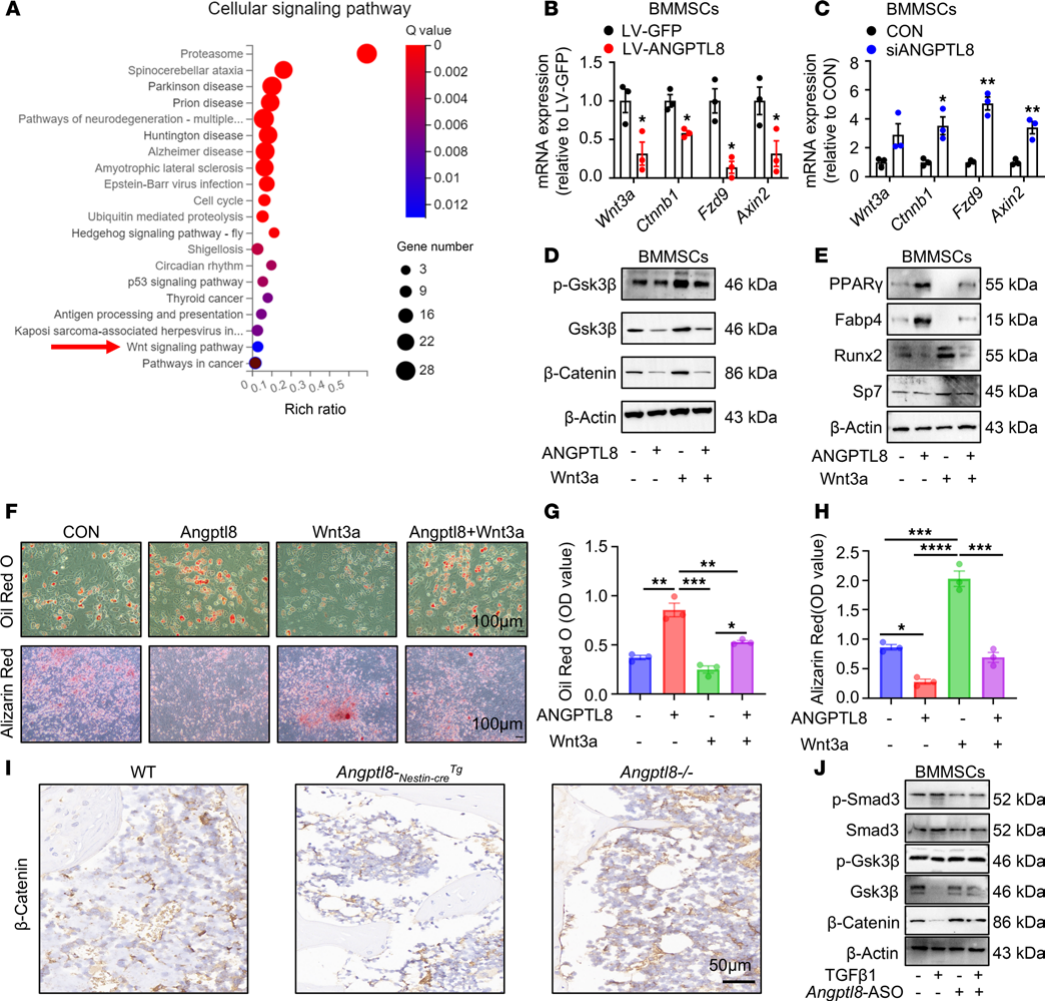
ANGPTL8 regulates MSC differentiation via the Wnt/β-catenin signaling pathway. (**A**) Pathway enrichment analysis in BMMSCs treated with LV-ANGPTL8. (**B**) qRT-PCR analysis of the mRNA expression of *Wnt3a*, *Fzd9*, *Axin2*, and *Ctnnb1* after BMMSC transfection with LV-GFP and LV-ANGPTL8. *n* = 3. (**C**) qRT-PCR analysis of the mRNA expression of *Wnt3a*, *Fzd9*, *Axin2*, and *Ctnnb1* after BMMSC transfection with siANGPTL8. *n* = 3. (**D**) ANGPTL8 blunted Wnt3a-induced phosphorylation of Gsk3β and prevented the subsequent accumulation of β-catenin in BMMSCs. *n* = 3. (**E**) Western blotting of PPARγ, Fabp4, Runx2, and Sp7 from BMMSCs treated with Angptl8 and Wnt3a. (**F**) Wnt3a rescued Oil Red O and alizarin red staining of lineage differentiation bias caused by overexpression of ANGPTL8 in BMMSCs. *n* = 3. Scale bars: 100 μm. (**G**) Quantification of Oil Red O staining in **F**. *n* = 3. (**H**) Quantification of calcium mineralization based on alizarin red staining in **F**. *n* = 3. (**I**) Immunohistochemical staining of β-catenin in the femurs of WT, *Angptl8-_Nestin-cre_Tg*, and *Angptl8^–/–^* mice. Scale bar: 50 μm. (**J**) Western blotting of p-Smad3, Smad3, p-Gsk3β, Gsk3β, and β-catenin from BMMSCs treated with TGF-β1 and *Angptl8*-ASO. Data are mean ± SEM. *P < 0.05; **P < 0.01; ***P < 0.001; ****P < 0.0001 by 2-tailed Student’s *t* test (**B** and **C**) or 2-way ANOVA followed by Tukey’s multiple-comparison test (**G** and **H**).

To elucidate the underlying mechanism of ANGPTL8 in BMMSCs, we performed mRNA sequencing to assess ANGPTL8-induced transcriptional changes. Pathway enrichment analysis indicated that pathways downregulated following LV-ANGPTL8 treatment were primarily associated with the Wnt signaling pathway (Figure 5A). Wnt/β-catenin signaling is a crucial pathway that regulates the differentiation of MSCs (26–29). As expected, ANGPTL8 inhibited the markers (such as *Wnt3a*, *Ctnnb*, *Fzd9*, and *Axin2*) of Wnt/β-catenin signaling in BMMSCs (Figure 5B) and hBMMSCs (Supplemental Figure 7A) after transfection with LV-ANGPTL8, while it upregulated the markers of Wnt/β-catenin signaling in BMMSCs (Figure 5C) and hBMMSCs after transfection with siANGPTL8 (Supplemental Figure7B). Correspondingly, markers of Wnt/β-catenin signaling were inhibited in BM of *Angptl8-_Nestin-cre_Tg* and *Angptl8-_Cagg-cre_Tg* mice (Supplemental Figure 7, C and E), whereas they were upregulated in BM of *Angptl8*^–/–^ mice (Supplemental Figure 7D). To further validate this hypothesis, we observed that ANGPTL8 suppressed Wnt3a-induced phosphorylation of Gsk3β and prevented the subsequent accumulation of β-catenin in BMMSCs (Figure 5D and Supplemental Figure 7, F–H). Moreover, the lineage differentiation bias caused by ANGPTL8 overexpression in BMMSCs was rescued upon Wnt3a treatment. Western blot analysis confirmed that Wnt3a administration led to decreased expression of adipogenic markers (PPARγ and Fabp4) and increased expression of osteogenic markers (Runx2 and Sp7) (Figure 5E and Supplemental Figure 7, I–L). Consistent with these findings, Oil Red O staining revealed a reduction in lipid droplets, while alizarin red staining indicated enhanced mineralized nodule formation following Wnt3a treatment (Figure 5, F–H). Immunohistochemical staining showed a decrease in β-catenin in BM of *Angptl8-_Nestin-cre_Tg* and an increase in β-catenin in BM of *Angptl8*^–/–^ mice (Figure 5I). The role of ANGPTL8 in regulating BMMSC differentiation via the Wnt/β-catenin pathway was further confirmed in hBMMSCs (Supplemental Figure 7M).

We have identified that TGF-β1 upregulated ANGPTL8 expression in MSCs during bone aging (Supplemental Figure 2, L and M), which subsequently inhibited the phosphorylation of Gsk3β and reduced levels of active Gsk3β and β-catenin (Figure 5J). This suppression of Wnt/β-catenin signaling was attenuated by treatment with an ANGPTL8 inhibitor (*Angptl8* antisense oligonucleotide, *Angptl8*-ASO; Figure 5J). Although ANGPTL8 is known to regulate lipid metabolism primarily through forming complexes with ANGPTL3 or ANGPTL4 (30, 31), our data showed that ANGPTL8 continued to inhibit p-Gsk3β, Gsk3β, and β-catenin even after treatment with ANGPTL3- or ANGPTL4-neutralizing antibodies (Supplemental Figure 7, N and O). Thus, ANGPTL8 modulates BMMSC differentiation by inhibiting the Wnt/β-catenin signaling pathway independently of complex formation with ANGPTL3 or ANGPTL4. Together, these data validate the notion that ANGPTL8 represses Wnt/β-catenin signaling to prime BMMSCs toward adipogenic differentiation.

### PPARγ inhibition partially downregulated ANGPTL8 expression and rescued the phenotype of ANGPTL8 overexpression in MSCs.

**Figure 6:**
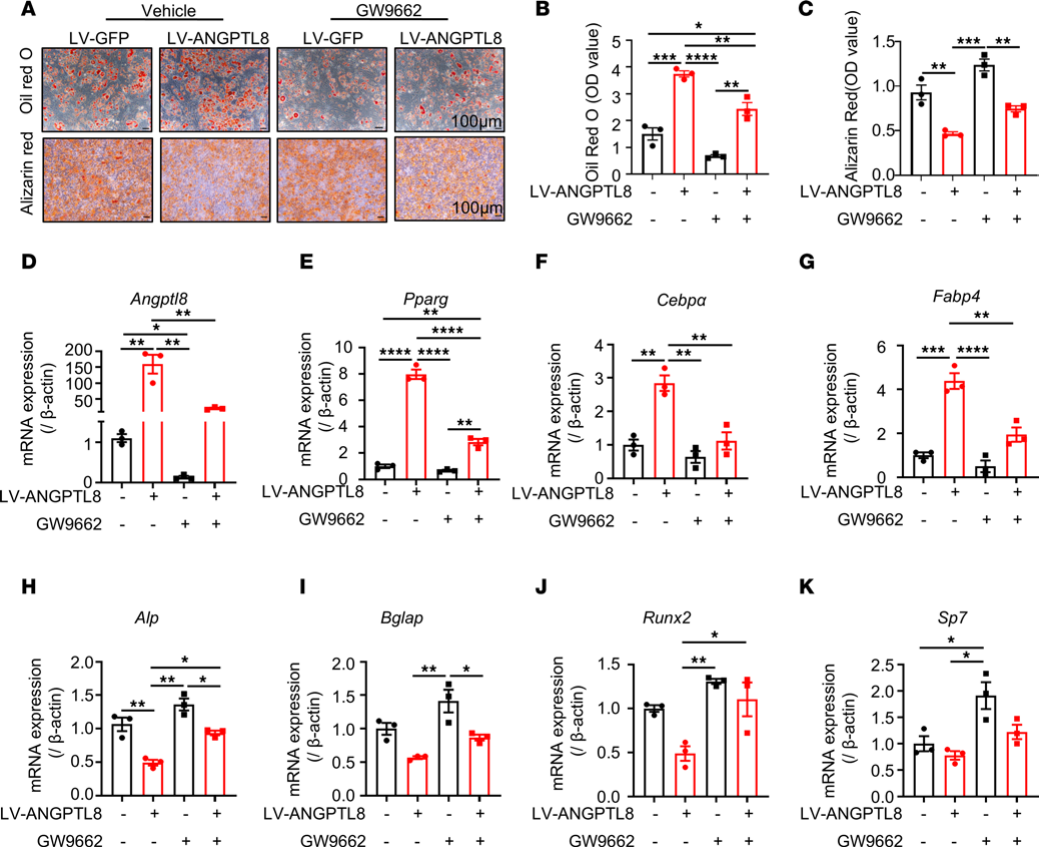
PPARγ inhibition partially inhibits ANGPTL8 expression and rescues the phenotype of ANGPTL8 overexpression in MSCs. (**A**) Representative images of Oil Red O staining and alizarin red staining in mouse BMMSCs after transfection with LV-GFP and LV-ANGPTL8 treated with vehicle or a PPARγ inhibitor (GW9662). Scale bars: 100 μm. (**B**) Quantification of Oil Red O staining in **A**. *n* = 3. (**C**) Quantification of calcium mineralization based on alizarin red staining in **A**. *n* = 3. (**D**) qRT-PCR analyses of the mRNA expression of *Angptl8* in GW9662-treated BMMSCs. (**E**–**G**) qRT-PCR analyses of the mRNA expression of *Pparg, Cebpα*, and *Fabp4* in GW9662-treated BMMSCs under adipogenic conditions. (**H**–**K**) qRT-PCR analyses of the mRNA expression of *Alp*, *Bglap*, *Runx2*, and *Sp7* in GW9662-treated BMMSCs under osteogenic conditions. *n* = 3 biologically independent BMMSC samples. Data are mean ± SEM. *P < 0.05; **P <0.01; ***P < 0.001; ****P < 0.0001 by 2-way ANOVA followed by Tukey’s multiple-comparison test.

PPARγ is a classic target to promote MSC differentiation into adipocytes (28, 29, 32). To confirm that activated PPARγ contributed to the pathogenic phenotype upon ANGPTL8 overexpression, we treated LV-ANGPTL8 BMMSCs with the PPARγ-specific antagonist GW9662. Following induction of adipogenesis, the decreased lipid droplets in the GW9662-treated LV-ANGPTL8 BMMSCs were evidenced by the diminished intensity of Oil Red O staining (Figure 6, A and B) and the mRNA levels of *Angptl8* were downregulated in these cells (Figure 6D), suggesting that GW9662 regulated ANGPTL8 expression. Meanwhile, decreased levels of traditional adipogenic genes (*Pparg*, *Cebpα*, and *Fabp4*) suggested that the adipogenic capacity of the GW9662-treated LV-ANGPTL8 BMMSCs was limited (Figure 6, E–G). Following osteogenic induction, an increase in alizarin red staining revealed the partial restoration of osteogenic differentiation in the GW9662-treated LV-ANGPTL8 BMMSCs (Figure 6, A and C). Moreover, the increased levels of osteogenic indicators *Alp*, *Bglap*, *Runx2*, and *Sp7* reinforced the restored osteogenic potency of these GW9662-treated cells (Figure 6, H–K). Subsequent studies in hBMMSCs (Supplemental Figure 8) and the cell line C3H10T1/2 (Supplemental Figure 9) confirmed these findings, leading to the conclusion that PPARγ inhibition partially downregulated ANGPTL8 expression and rescued the phenotype of ANGPTL8 overexpression in MSCs.

### Administration of a PPARγ inhibitor partially rescued the phenotype of Angptl8-_Nestin-cre_Tg mice.

**Figure 7:**
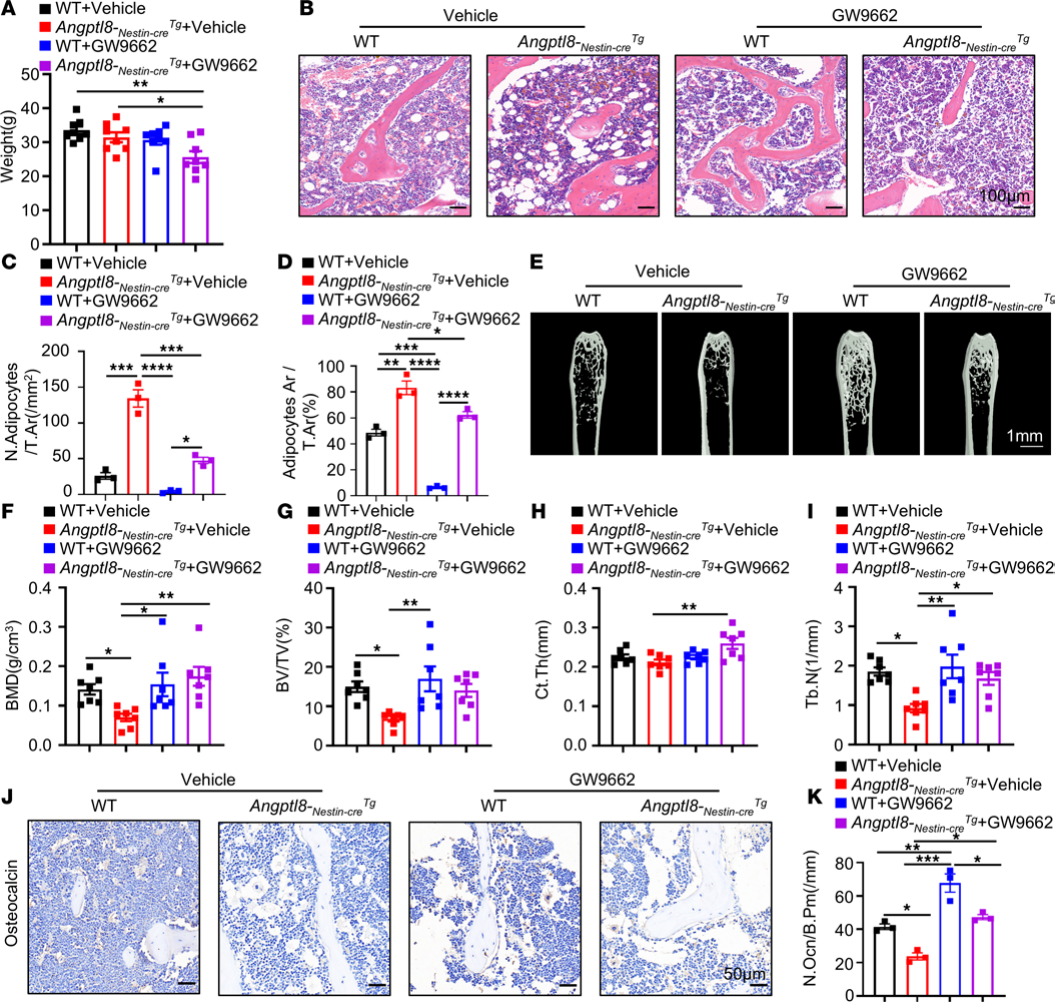
Administration of a PPARγ inhibitor partially rescues the phenotype of *Angptl8-_Nestin-cre_Tg* mice. (**A**) Weight of the GW9662-treated WT and *Angptl8-_Nestin-cre_Tg* male mice at 18 months. *n* = 7–8. (**B**) H&E staining the femur of the GW9662-treated WT and *Angptl8-_Nestin-cre_Tg* male mice at 18 months. Scale bars: 100 μm. (**C** and **D**) Quantification of number (**C**) and area (**D**) of adipocytes in the GW9662-treated WT and *Angptl8-_Nestin-cre_Tg* male mice at 18 months. *n* = 3. (**E**) Representative microCT images of distal femurs and midshaft cortical bone from the GW9662-treated WT and *Angptl8-_Nestin-cre_Tg* male mice at 18 months. Scale bar: 1 mm. (**F**–**I**) Quantitative microCT analyses of the distal end of the femurs from the GW9662-treated WT and *Angptl8-_Nestin-cre_Tg* male mice at 18 months. *n* = 7–8. BMD, bone mineral density; BV/TV, bone volume per tissue volume; Ct.Th, cortical bone thickness; Tb.N, trabecular number. (**J**) Representative osteocalcin-positive cell images of distal femurs from the GW9662-treated WT and *Angptl8-_Nestin-cre_Tg* male mice at 18 months. Scale bars: 50 μm. (**K**) Quantification of osteocalcin^+^ cells on the bone surface (number of osteocalcin^+^ cells per bone perimeter, N.Ocn^+^/B.Pm). Data are mean ± SEM. *P < 0.05; **P < 0.01; ***P < 0.001; ****P < 0.0001 by 2-way ANOVA followed by Tukey’s multiple-comparison test.

To further investigate whether PPARγ inhibitor administration could alleviate the phenotype of *Angptl8-_Nestin-cre_Tg* mice, the PPARγ inhibitor GW9662 was injected into the BM cavity of 15-month-old *Angptl8-_Nestin-cre_Tg* mice once a week for a period of 3 months. Injection of GW9662 partly recovered the weight of the *Angptl8-_Nestin-cre_Tg* mice (Figure 7A). H&E staining validated the inhibited BMAT development in GW9662-treated *Angptl8-_Nestin-cre_Tg* mice (Figure 7, B–D). MicroCT analysis of the femur metaphysis revealed partially improved trabecular and cortical bone in GW9662-treated *Angptl8-_Nestin-cre_Tg* mice, as displayed by enhanced skeletal parameters, e.g., BMD, BV/TV, Ct.Th, and Tb.N (Figure 7, E–I). And then there were more osteocalcin-positive cells in distal femurs from the treated *Angptl8-_Nestin-cre_Tg* mice (Figure 7, J and K). These results suggested administration of a PPARγ inhibitor partially rescued the phenotype of *Angptl8-_Nestin-cre_Tg* mice.

### Angptl8-ASO rejuvenated the phenotype of aging mice.

**Figure 8:**
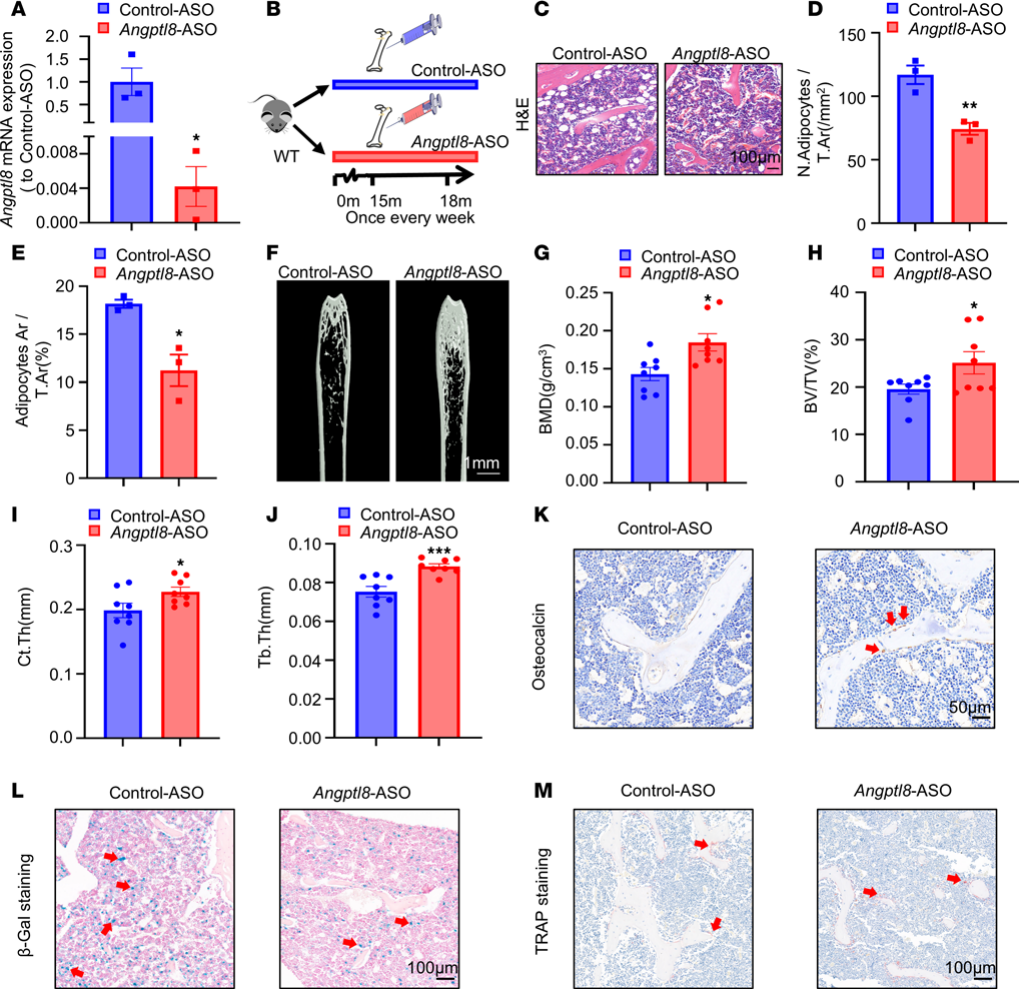
Administration of *Angptl8*-ASO partially rescues the phenotype of aging mice. (**A**) qRT-PCR analyses of the mRNA expression of *Angptl8* in the BMMSCs transfected with control-ASO and *Angptl8*-ASO. *n* = 3. (**B**) Experimental scheme to demonstrate that *Angptl8*-ASO partially rescues the phenotype of aging male mice. (**C**) H&E staining of the femurs of the control-ASO and *Angptl8*-ASO male mice at 18 months. Scale bar: 100 μm. *n* = 8. (**D** and **E**) Quantification of number (**D**) and area (**E**) of adipocytes in the control-ASO and *Angptl8*-ASO male mice at 18 months. *n* = 3. (**F**) Representative microCT images of distal femurs and midshaft cortical bone from the control-ASO and *Angptl8*-ASO male mice at 18 months. Scale bar: 1 mm. *n* = 8. (**G**–**J**) Quantitative microCT analyses of the distal end of the femurs from the control-ASO and *Angptl8*-ASO male mice at 18 months. *n* = 8. BMD, bone mineral density; BV/TV, bone volume per tissue volume; Ct.Th, cortical bone thickness; Tb.Th, trabecular thickness. (**K**) Representative osteocalcin^+^ cell images of distal femurs from the control-ASO and *Angptl8*-ASO male mice at 18 months. Scale bar: 50 μm. *n* = 8. (**L**) Representative β-Gal staining images of distal femurs from the control-ASO and *Angptl8*-ASO male mice at 18 months. Scale bar: 100 μm. *n* = 8. (**M**) Representative TRAP staining images of distal femurs from the control-ASO and *Angptl8*-ASO male mice at 18 months. Scale bar: 100 μm. *n* = 8. Data are mean ± SEM. *P < 0.05; **P < 0.01; ***P < 0.001 by 2-tailed Student’s *t* test.

To investigate the potential therapeutic benefits of BMMSC-specific inhibition of ANGPTL8 on age-related phenotypes, we first examined the effect of *Angptl8*-ASO on *Angptl8* expression in BMMSCs from WT male mice in vitro. Treatment with *Angptl8*-ASO substantially reduced *Angptl8* mRNA levels (Figure 8A). Subsequently, 15-month-old C57BL/6J male mice were administered either *Angptl8*-ASO or control-ASO at a dosage of 25 mg/kg body weight weekly for 3 months (Figure 8B). H&E staining confirmed a reduction in both the number and size of adipocytes in *Angptl8*-ASO–treated mice compared with the control-ASO group (Figure 8, C–E). MicroCT analysis of the femoral metaphysis indicated a partial recovery of trabecular and cortical bone architecture in *Angptl8*-ASO–treated mice, as demonstrated by improvements in BMD, BV/TV, Ct.Th, and trabecular thickness (Tb.Th) (Figure 8, F–J). Furthermore, mice receiving *Angptl8*-ASO exhibited an increase in osteoblast numbers and a reduction in senescent cells relative to control-treated animals (Figure 8, K and L). In contrast, osteoclast number remained unchanged following *Angptl8*-ASO treatment (Figure 8M). These results suggested that old mice treated with *Angptl8*-ASO exhibited rejuvenated bone metabolic health.

## Discussion

Our research showed a direct link between BMAT expansion and ANGPTL8, as well as a negative association between ANGPTL8 and skeletal health in various aging mouse models, including *Angptl8-_Nestin-cre_Tg*, *Angptl8-_Cagg-cre_Tg*, and *Angptl8*^–/–^ mice. TGF-β1 upregulated ANGPTL8 in BMMSCs during bone senescence. Subsequently, ANGPTL8 enhanced the expression of PPARγ while concurrently inhibiting the Wnt/β-catenin signaling pathway during the regulation of BMMSC differentiation. Notably, inhibition of the Wnt/β-catenin pathway itself contributed to the upregulation of PPARγ. *Angptl8*-ASO rejuvenated the phenotype of aging mice. Our results position ANGPTL8 as a crucial controller of BM homeostasis, functioning as an overlooked link between BMAT and skeletal wellness (Supplemental Figure 10).

Adipokines have diverse functions in regulation of bone remodeling. For instance, leptin is known to be beneficial for bone health, as a lack of it has been linked to decreased bone growth (33) and restoring it brings bone density back to normal (34). Conversely, adiponectin has been found to impede osteoblast proliferation and encourage apoptosis (35). Surprisingly, the role of ANGPTL8 in BM is frequently disregarded even though it is synthesized by the liver and adipose tissue. It is well documented that BMAT expands in various pathophysiological conditions (36, 37), including metabolic stress (38, 39). We demonstrated that increased ANGPTL8 production from MSCs in BM during skeletal aging promoted further BMAT development at the cost of bone formation. Through this ANGPTL8-mediated process, BM could promptly adapt to aging changes. Therefore, a mechanism is suggested to uphold plasticity in BM through ANGPTL8. Our study contributes to a deeper understanding of BMAT development and the bidirectional connection between bone wellness and BMAT, aspects that have not been adequately explored by investigated adipokines.

The production of ANGPTL8 by the liver and peripheral adipose tissue has been extensively studied. However, no previous study to our knowledge has emphasized the latent role of the BM as a vital source of ANGPTL8. The unique microenvironment provided by the BM is significantly influenced by the biology of BMAT, which is recognized as a distinctive fat storage site with unique function, localization, and regulation (40–43). Our studies revealed that MSCs from BM were one of the sources of ANGPTL8. In addition, after overexpression of ANGPTL8, MSCs had an increased ability for adipogenesis even following PPARγ activation, underscoring a direct impact of ANGPTL8 on the BM niche in vivo. Conversely, the absence of ANGPTL8 protected against bone loss in both MSCs and mice.

Adipsin has been shown to promote BMAT formation and affect skeletal remodeling in BM by curbing the Wnt/β-catenin signaling pathway (26). The Wnt ligand blocks Gsk3β activity in the canonical the Wnt/β-catenin signaling pathway by phosphorylating it, which stops β-catenin phosphorylation. Due to this, β-catenin is able to move into the nucleus and facilitate the transcription of target genes located downstream (28, 44). This pathway is well established as inhibiting adipogenesis by downregulating PPARγ expression and promoting bone formation by upregulating the expression of RUNX2 (45–47), the principal osteogenic transcription factor. Based on the effects of ANGPTL8 on the overall microenvironment of BM observed in our study, we found that in BMMSCs and hBMMSCs, β-catenin and Gsk3β were reduced and the phosphorylation of Gsk3β was inhibited under the influence of ANGPTL8. Although the addition of Wnt3a could promote the expression of β-catenin, Gsk3β, and the phosphorylation of Gsk3β, these effects could still be inhibited by ANGPTL8, indicating that ANGPTL8 could suppress the Wnt/β-catenin signaling pathway to influence the fate of MSCs.

In previous studies, it was found that PPARγ functioned as a crucial nuclear receptor that controlled the differentiation of MSCs from BM and their fate choices (48). The methylation and acetylation of histone lysine residues, in concert with its interactions with coactivators and corepressors, constitute the underlying mechanism (49). MSCs from BM possess a multidirectional differentiation capacity that primarily commits to osteogenic, adipogenic, and chondrogenic lineages. Activation of PPARγ predominantly drives MSC fate from BM toward adipogenesis (49, 50). However, by utilizing the inhibitory effect of PPARγ on certain precursor cytokines or other transcriptional inhibitors, the cell fate of MSCs from BM favoring adipogenic differentiation can also be redirected toward osteogenic differentiation (50, 51). In accordance with our findings, the increased PPARγ signaling pathway aligned with the phenotype of ANGPTL8-overexpressing MSCs from BM in vivo and in vitro. Moreover, the administration of a PPARγ inhibitor largely shifted MSCs from BM from adipogenic differentiation to predominantly osteogenic differentiation, further confirming the PPARγ signaling pathway as a mechanism by which ANGPTL8 influences cell lineage allocation in MSCs from BM.

Elucidating the determinants that regulate ANGPTL8 expression represents a promising research direction. We previously revealed that insulin upregulated ANGPTL8 in hepatocytes (52); however, it is possible that other factors also affect ANGPTL8 expression. A hallmark of aging is the accumulation of senescent cells, which secrete a distinct profile of factors that thereby modify the tissue milieu (53). The concept of the senescence-associated secretory phenotype (SASP) was introduced in 2008 by Coppe and colleagues, stemming from their quantitative profiling of factors secreted by senescent cells. This phenotype encompasses cytokines, chemokines, growth factors, and proteases (54, 55). A recent investigation demonstrated that eliminating senescent cells can halt the bone loss associated with aging (56). Our studies found that TGF-β1 at least partially contributed to increased *Angptl8* expression by phosphorylating Smad3 in MSCs during aging, supporting the conclusion that aging leads to increased ANGPTL8 levels. However, whether ANGPTL8 in MSCs is influenced by the other SASP of aging cells in BM warrants further investigation.

The hallmark of cellular senescence — an irreversible cessation of proliferation — is orchestrated by the key p16INK4A/Rb and p19ARF/p53 signaling pathways (53, 57–59). A significant discovery from our research was that ANGPTL8 promoted MSC aging through regulation of *p16INK4A* gene expression. In summary, the relationship between aging and ANGPTL8 forms a positive feedback loop. Notably, ANGPTL8 overexpression has been linked to various cancers (60, 61). Since senescence has the potential to safeguard cells from cancer progression (59, 62, 63), our findings may provide insight into ANGPTL8-associated mechanisms of tumorigenesis. Further research is necessary to investigate the safeguarding role of the ANGPTL8/p16 cascade in preventing premature senescence or uncontrolled proliferation in various cell types.

Our findings demonstrate that ANGPTL8 functions as a priming molecule for MSCs, which aids in clarifying why aged mice display bone loss and elevated BMAT phenotypes. This function of ANGPTL8 may offer a new approach for treating bone loss in elderly patients. Evidence from prior studies indicates that *Angptl8*-ASO treatment can ameliorate lipid metabolism and protect against high-fat diet–induced NAFLD in animal models (64, 65). We discovered that *Angptl8*-ASO ameliorated the phenotype of aging mice. Therefore, we speculate that *Angptl8*-ASO may have applications in treating age-related osteoporosis. Hence, based on our findings, these current therapies show promise in being repurposed for the treatment and prevention of age-related osteoporosis.

*Limitations of the study*. Regarding the source of ANGPTL8, while studies using ANGPTL8-overexpressing mice strongly support a BMMSC-autonomous role, systemic interventions such as ANGPTL8 knockout and *Angptl8*-ASO do not fully resolve this issue due to their whole-body effects. Since loss-of-function strategies appear to be therapeutically relevant, using *Angptl8*-ASO to attenuate BMD loss in *Angptl8-_Nestin-cre_Tg* mice may represent an optimal experimental approach. Furthermore, once the in vivo expression pattern of ANGPTL8 is clarified, appropriate transgenic mouse models should be established. For example, employing a BMMSC-specific Cre driver (e.g., Prx1-Cre) would help delineate the skeletal-specific functions of ANGPTL8 and reduce confounding effects from other tissues, as observed in systemic *Angptl8^–/–^* models. Finally, bulk or single-cell RNA sequencing of these transgenic models has not yet been conducted to identify upstream and downstream regulators of ANGPTL8. Our research group will continue to investigate this question in greater depth.

### Conclusion

ANGPTL8 modulates MSC fate in the course of skeletal aging and exerts an impact on skeletal remodeling within the BM niche. Our research uncovers a mechanism through which the BM maintains its intrinsic plasticity via the endocrine effects of a distinct adipokine, ANGPTL8.

## Methods

### Sex as a biological variable.

Male mice and individuals were used in this study. We examined male mice and individuals because this ruled out the effect of estrogen on bone phenotype.

### Mice.

Our care for the animals followed the guidelines specified in the Manual for Laboratory Animal Care and Use, which was compiled by the National Academy of Sciences and released by the NIH. WT C57BL/6J mice were obtained from Beijing Huafukang Bioscience Co., Ltd., while all transgenic mice were produced and obtained from Cyagen Biosciences Inc. The rodents were provided with free access to food and water and were housed in a controlled environment with a 12-hour/12-hour light/dark cycle at a temperature of 23°C ± 2°C. They were fed a standard diet (10% fat, 70% carbohydrate, and 20% protein; Jiangsu Xietong, Inc., SWS9102). At the end of the study, mice were humanely euthanized using carbon dioxide asphyxiation, followed by cervical dislocation to ensure complete euthanasia, after which tissues were collected for analysis.

### Study population.

Samples of human BM were acquired from patients who were undergoing orthopedic procedures at Tongji Hospital of Huazhong University of Science and Technology. These patients with femoral neck or femoral head fractures experiencing hip joint replacement were included in the study. All participants underwent detailed screening using a questionnaire, medical history, and physical examination. Individuals were excluded if they had any conditions that could impact bone metabolism, including kidney, liver, parathyroid, and thyroid disorders, diabetes, hyperprolactinemia, rheumatoid arthritis, ankylosing spondylitis, malabsorption syndromes, cancer, blood disorders, or had suffered pathological fractures in the past 12 months. Informed written consent was obtained from all patients.

### Rescue strategy in vitro and in vivo.

Based on the verification of the PPARγ signaling pathway, MSCs were treated with the PPARγ-specific antagonist GW9662 (10 μM, dissolved in DMSO; MedChemExpress) to rescue the phenotype in vitro. Intra-BM delivery of inhibitor or ASO was performed as previously reported (8). To partially rescue the general body phenotype in vivo, we injected 3 mg/kg/week GW9662 into the male *Angptl8-_Nestin-cre_Tg* mice and control littermates by intra-BM delivery until 12 weeks old before sacrifice.

Mice were treated with *Angptl8*-ASO or a control-ASO that does not target any identified rat, mouse, or human gene. The ASO sequences were as follows: *Angptl8*-ASO (5′-TTTCTGTACAGTGTCATGTGC-3′) and control-ASO (5′-AGCATAGTTAACGAGCTCCC-3′) with underlined sequences indicating 2′-O-methoxyethyl–modified bases. To partially rescue the aging phenotype in vivo, we injected 25 mg/kg/week *Angptl8*-ASO or a control-ASO into the old male mice by intra-BM delivery until 12 weeks old before sacrifice.

### Cell cultures.

Mouse BMMSCs were isolated according to previously established methods (66). In summary, tibias and femurs were harvested from mice, crushed, and digested using collagenase A (MilliporeSigma) to create a single-cell suspension. The cells in the resulting liquid were then exposed to PE-, APC-, FITC-, and BV421-conjugated antibodies specific for mouse Sca-1 (catalog 108107), CD29 (catalog 102215), CD45 (catalog 103108), and CD11b (catalog 101235) from BioLegend at low temperatures for 20 minutes. Analysis was carried out with a FACSAria flow cytometer from BD Biosciences and FACSDiva software version 6.1.3. The sorted cells were then cultured until they reached 80%–85% confluence, after which first-passage MSCs were separated and plated for cell population enrichment. Adipogenic and osteogenic differentiation was performed only with third-passage MSCs. The cell line C3H10T1/2 was obtained from ATCC.

### Adipogenic differentiation assay.

To induce adipogenic differentiation in vitro, MSCs were transfected with siANGPTL8 or LV-ANGPTL8 (Genechem) and then seeded in 6-well plates at a density of 2.5 × 10^6^ cells per well. The cells were cultured in α-MEM supplemented with 10% fetal bovine serum (FBS), 0.5 mM 3-isobutyl-1-methylxanthine, 5 μg/mL insulin, and 1 μM dexamethasone for a period of 14 days. The culture medium was refreshed every alternate day. The presence of lipid droplets in mature adipocytes was visualized using Oil Red O staining (Cyagen).

Osteogenic differentiation assay. For the differentiation of osteoblasts, MSCs were transfected with siANGPTL8 or LV-ANGPTL8 and then seeded in 24-well plates at a density of 5 × 10^5^ cells/well. The cells were cultured in α-MEM supplemented with 10% FBS, 0.1 mM dexamethasone, 10 mM β-glycerol phosphate, and 50 mM ascorbate-2-phosphate for a period of 14 days. Following this, mineralization of the cell matrix was assessed by either staining with 2% alizarin red (Cyagen) at pH 4.2 or using an ALP staining kit (Beyotime). Imaging was performed using a Diaphot Inverted Microscope and Camera System (Olympus).

### Cellular senescence assay.

C3H10T1/2 were plated in 6-well dishes at a density of 1.0 × 10^6^ cells per well and incubated for 24 hours. Aging cells were detected by performing β-Gal staining, utilizing a kit from Beyotime, following the provided guidelines.

### Antibody blockade assay.

Mouse BMMSCs were pretreated with anti-ANGPTL3 antibody (10 μg/mL; MedChemExpress, HY-P99194) or anti-ANGPTL4 antibody (10 μg/mL; Selleck, A2772) for 24 hours before transfection with LV-ANGPTL8 for 24 hours to detect Wnt/β-catenin signaling pathway.

### RNA sequencing.

RNA was extracted from the treated BMMSCs, and the samples were sent to MGI Tech Co., Ltd. for mRNA sequencing. Gene expression analysis was performed by mRNA sequencing on a BGI-SEQ500 (MGI Tech Co.). The RNA-seq data have been deposited in the NCBI Sequence Read Archive database under accession code PRJNA1314422.

### qRT-PCR.

RNA was extracted using TRIzol reagent (Vazyme), and cDNA was synthesized by the GoScript Reverse Transcription System (Vazyme). qRT-PCR was conducted on the ABI 7500 system (Applied Biosystems) utilizing SYBR Green (Vazyme). The primers for qRT-PCR are displayed in Supplemental Table 1.

### Histochemistry analysis.

Histochemical analysis was conducted in accordance with established protocols (9, 61). Following euthanasia, bones were harvested and fixed in 4% paraformaldehyde (PFA) at 4°C for 24 hours, subsequently decalcified in 10% EDTA for a period of 3 weeks at 4°C, and finally embedded in paraffin. Longitudinal sections of bone measuring 4 μm thick were stained with TRAP and H&E (Servicebio) as per the guidelines provided by the manufacturer.

### MicroCT analysis.

The bone underwent microCT scanning with a SkyScan 1176 (Bruker) device. Using the CTAn software, a 3D representation of the bone was created, and various structural parameters were determined. The specific area analyzed was chosen as 5 mm beneath the bone’s growth plate.

### ELISA.

Commercially available mouse ELISA kits were used to measure ANGPTL8 (EIAab Science Inc., E11644m), TGF-β1 (Record Biotechnology, 02289M2), TGF-β2 (Record Biotechnology, 02418M2), and TGF-β3 (Record Biotechnology, 12416M2) levels in the serum or cell supernatants according to the manufacturer’s instructions. Commercially available human ELISA kits were used to measure ANGPTL8 (EIAab Science Inc., E11644h), TGF-β1 (Record Biotechnology, 03245H2), TGF-β2 (Record Biotechnology, 03426H2), and TGF-β3 (Record Biotechnology, 05192H2) levels in the BM or cell supernatants according to the manufacturer’s instructions.

### Immunofluorescent staining.

MSCs in culture were fixed using 4% PFA for 15 minutes at ambient temperature. Next, the cells underwent blocking with 5% bovine serum albumin (BSA) for 1 hour at room temperature, followed by overnight incubation at 4°C with anti-ANGPTL8 antibody (Biorbyt, orb544742; 1:100) and anti-PPARγ antibody (Cell Signaling Technology, 2443; 1:100). Afterwards, the cells were exposed to secondary antibodies conjugated with Alexa Fluor 488 (Invitrogen, A21106) and Alexa Fluor 555 (Invitrogen, A21422). Finally, the nuclei were counterstained utilizing DAPI.

### Immunohistochemical staining.

The immunohistochemical staining procedure followed previously published methods (9, 61). Following antigen retrieval, bone sections underwent blocking with 5% BSA for 1 hour at room temperature and were then incubated overnight at 4°C with primary antibodies targeting osteocalcin (Servicebio, GB11233-100) or β-catenin (Servicebio, GB12015-100). Subsequently, sections were treated with the secondary antibody at room temperature for 1 hour. Detection of immunoreactivity was achieved using an HRP-streptavidin system (Dako), and the slides were afterwards counterstained with hematoxylin.

### Calcein double-labeling assay.

To evaluate dynamic bone formation ability, mice were administered intraperitoneally with calcein (25 mg/kg, Sigma-Aldrich) at 14 and 7 days before euthanasia. After fixation in 70% ethanol, the samples were dehydrated in gradient ethanol. Then, the calcein-double-labeled bones were embedded in methyl methacrylate. Longitudinal bone sections (5 μm thick) were made using a microtome and observed under a fluorescence microscope. BFR and MAR were measured using OsteoMeasureXP software (Osteo-Metrics, Inc.). MAR and BFR can be measured directly. MAR is the rate of formation of mineralized layer on the surface of trabecular bone, which is calculated by dividing the distance between 2 markers by the interval time between markers. BFR: The length of the tetracycline labels (mineralizing surface per bone surface [MS/BS]) multiplied by the distance between labels (MAR) is the area of new bone formed during the label interval; thus, BFR = MS/BS × MAR (67).

### Western blot.

Cell lysis was performed with NP40 lysis buffer (Beyotime) supplemented with protease and phosphatase inhibitor cocktails (Selleck). Following separation by SDS-PAGE, protein samples were transferred to a nitrocellulose membrane (Bio-Rad), blocked in 5% nonfat milk in TBST, and probed with primary antibodies against ANGPTL8 (Biorbyt, orb544742; 1:1000), PPARγ (Cell Signaling Technology, 2443; 1:1000), Fabp4 (Proteintech, 12802; 1:1000), p21 (Santa Cruz Biotechnology Inc., sc-150; 1:500), Runx2 (Abcam, ab13979; 1:1000), Sp7 (Abcam, ab13979; 1:1000), p16 (Santa Cruz Biotechnology, sc-1661; 1:500), p-Gsk3β (Abclonal, AP1088; 1:1000), Gsk3β (Abclonal, A11731; 1:1000), Smad3 (Abclonal, A19115; 1:10000), p-Smad3 (Abclonal, AP0727; 1:1000), ANGPTL3 (Abclonal, A5225; 1:1000), ANGPTL4 (Abclonal, A2011; 1:1000), Fasn (Abclonal, A5225; 1:1000), Ldlr (Proteintech, 66414; 1:1000), and β-actin (Proteintech, 66009; 1:8000) overnight at 4°C. Protein detection was achieved with HRP-conjugated secondary antibody and chemiluminescent HRP substrate (Proteintech).

### Three-point bending test.

The tibial cortical strength at the midshaft was assessed using a 3-point bending examination performed on a 3WDW3100 mechanical-testing apparatus (Nuochen Biotechnology) fitted with a 500 N M-SI transducer by Celtron Technologies Inc. This assessment involved 2 terminal support points and a single central loading point, with the span length between the 2 support points set to 60% of the entire bone length. Each bone was subjected to loading at a consistent velocity of 0.155 mm/s until reaching failure. Biomechanical data were extracted from the load-deformation curves, with the maximum load (in N) and stiffness (in N/mm) being documented.

### Statistics.

Data are presented as mean ± SEM. Comparisons between 2 groups utilized 2-tailed Student’s *t* tests, while multiple comparisons (3 or more groups) were analyzed using 1- or 2-way ANOVA with Tukey’s post hoc test. Any *P* value of less than 0.05 was deemed statistically significant.

### Study approval.

All experiments involving animals were conducted according to the ethical policies and procedures approved by the Institutional Animal Care and Use Committee of Tongji Hospital, Tongji Medical College, Huazhong University of Science and Technology, China (approval no. TJH-202009014).

All experiments involving human samples were conducted according to the ethical policies and procedures approved by the ethics committee of Tongji Hospital, Tongji Medical College, Huazhong University of Science and Technology, China (approval no. TJ-IRB20230225).

### Data availability.

Values for all data points in graphs are reported in the Supporting Data Values file. The RNA sequencing data generated in this study have been deposited in the NCBI Sequence Read Archive database under accession code PRJNA1314422. The data that support the findings of this study are also available from the corresponding author upon reasonable request.

## Author contributions

XY conceptualized the syudy. YG, ZZ, and JH curated data. YG and ZZ analyzed data. XY acquired funding. YG conducted experiments. PL and ZW developed methodology. XY, YC, and DL provided project administration. XY, FG, HY, and FL provided resources. YZ, XM, RK, and SW provided software. XY supervised the study. YG validated results. BM, XX, LP, YH, and YY generated figures. YG, YC, and XY wrote, reviewed, and edited the manuscript.

## Funding support

National Natural Science Foundation of China grants 82470907, 82270880, 81974109 (to XY) and 82350610277 (to YC).

## Supplementary Material

Supplemental data

Unedited blot and gel images

Supporting data values

## Version 1 10/21/2025

In-Press Preview

## Version 2 12/08/2025

Electronic publication
