# Supplementary material for: Angiopoietin-like 8 governs osteoblast-adipocyte lineage commitment during skeletal aging
Source: JCI Insight. 2025 Oct 21;10(23):e189371. doi: 10.1172/jci.insight.189371 (PMC12890479; doi:10.1172/jci.insight.189371)
Supplement: Supplemental data [file jciinsight-10-189371-s139.pdf]

Supplemental Figure of

Angiopoietin-like protein 8 governs osteoblast-adipocyte lineage commitment during skeletal aging

Authors Names: Yaming Guo[1,2,3,5], Zeqing Zhang[1,2,3,5], Junyu He[1,2,3], Peiqiong Luo[1,2,3], Zhihan Wang[1,2,3], Yurong Zhu[1,2,3], Xiaoyu Meng[1,2,3], Limeng Pan[1,2,3], Ranran Kan[1,2,3], Yuxi Xiang[1,2,3], Beibei Mao[1,2,3], Yi He[1,2,3], Siyi Wang[1,2,3], Yan Yang[1,2,3], Fengjing Guo[4], Hongbo You[4], Feng Li[4], Danpei Li[1,2,3#],Yong Chen[1,2,3#], Xuefeng Yu[1,2,3#]

1.Division of Endocrinology, Department of Internal Medicine, Tongji Hospital, Tongji Medical College, Huazhong University of Science and Technology, Wuhan, China.

2.Hubei Clinical Medical Research Center for Endocrinology and Metabolic Diseases, Hubei, China.

3.Branch of National Clinical Research Center for Metabolic Diseases, Hubei, China.

4.Department of Orthopedics, Tongji Hospital, Tongji Medical College, Huazhong University of Science and Technology, Wuhan 430030, China.

5.Contribution equally.

#Corresponding author:

Danpei Li, M.D., Ph.D., Division of Endocrinology, Department of Internal Medicine, Tongji Hospital, Tongji Medical College, Huazhong University of Science and Technology, 1095 Jiefang Avenue, Wuhan 430030, China.

Email: ldp19940730@163.com.

Yong Chen, M.D., Ph.D., Professor of Medicine, Division of Endocrinology, Department of Internal Medicine, Tongji Hospital, Tongji Medical College, Huazhong University of Science and Technology, 1095 Jiefang Avenue, Wuhan 430030, China.

Email: tj.y.chen@vip.163.com.

Xuefeng Yu, M.D., Ph.D., Professor of Medicine, Division of Endocrinology, Department of Internal Medicine, Tongji Hospital, Tongji Medical College, Huazhong University of Science and Technology, 1095 Jiefang Avenue, Wuhan 430030, China.

Email: xfyu188@163.com.

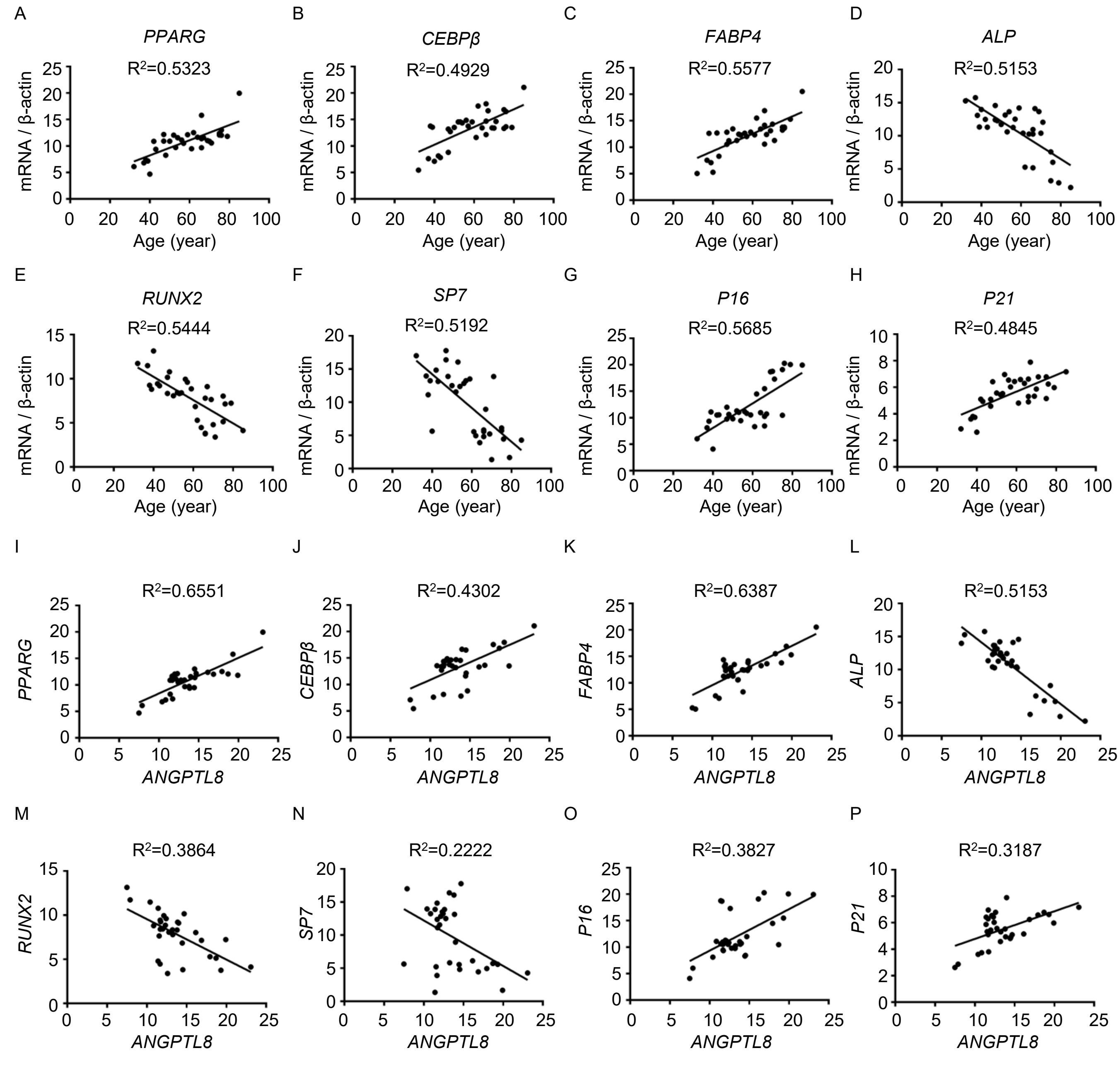
A
PPARG
R2=0.5323
B
CEBPβ
R2=0.4929
C
FABP4
R2=0.5577
D
ALP
R2=0.5153
E
RUNX2
R2=0.5444
F
SP7
R2=0.5192
G
P16
R2=0.5685
H
P21
R2=0.4845
mRNA / β-actin
Age (year)
I
R2=0.6551
J
R2=0.4302
K
R2=0.6387
L
R2=0.5153
M
R2=0.3864
N
R2=0.2222
O
R2=0.3827
P
R2=0.3187
PPARG
CEBPβ
FABP4
ALP
RUNX2
SP7
P16
P21
ANGPTL8

Supplemental Figure 1. ANGPTL8 expression increased with age in bone marrow during skeletal aging.

(A-P) Association of mRNA expression of *PPARG*、 *CEBPβ*、 *FABP4*、*ALP*、*RUNX2*、*SP7*、 *P16 and P21* in human BMMSCs from individuals with ages (n = 33) (A-H) and with *ANGPTL8* mRNA expression (n = 33) (I-P).

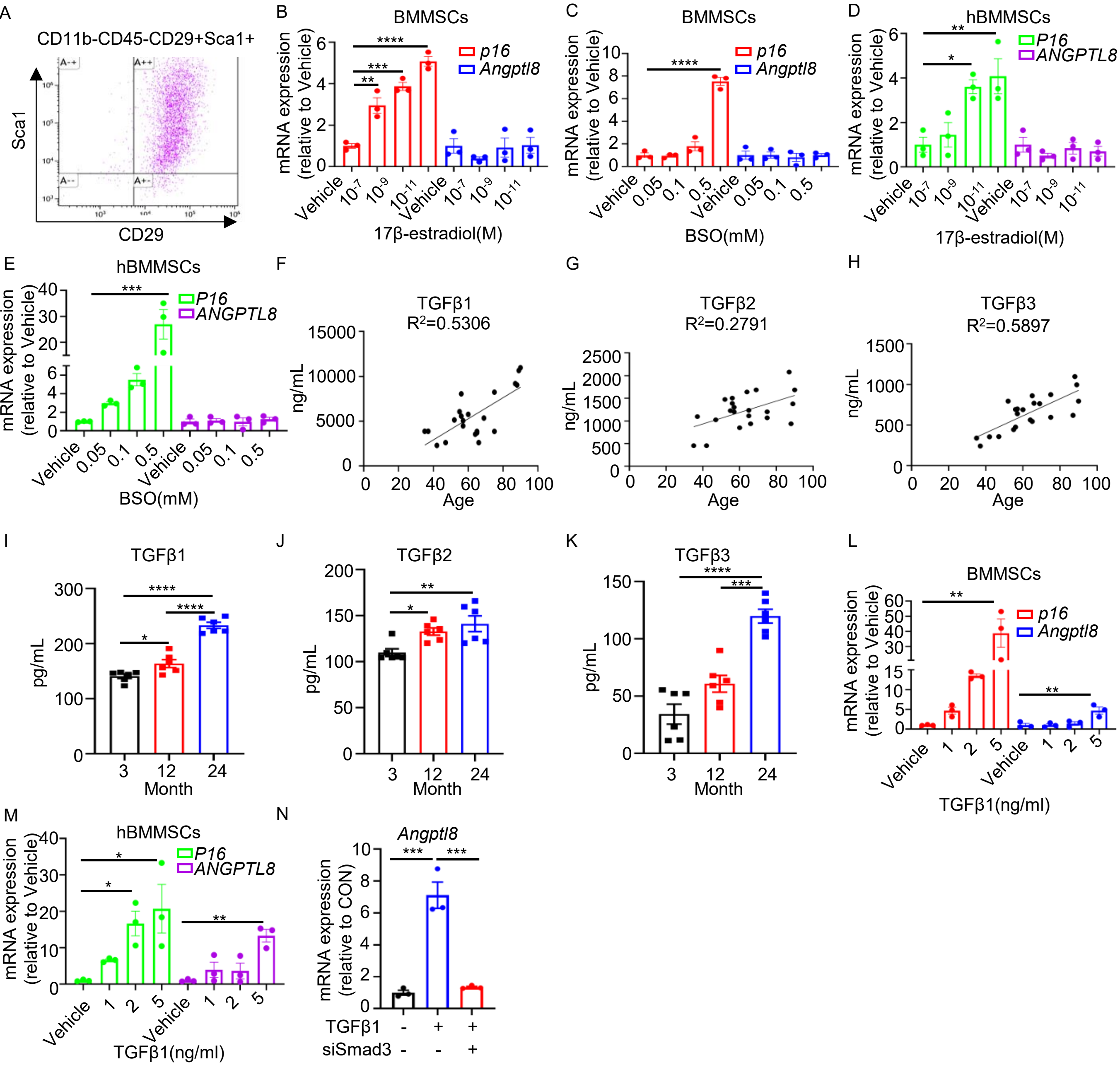
A
CD11b-CD45-CD29+Sca1+
Sca1
CD29
B
BMMSCs
p16
Angptl8
mRNA expression (relative to Vehicle)
17β-estradiol(M)
C
BMMSCs
BSO(mM)
D
hBMMSCs
P16
ANGPTL8
17β-estradiol(M)
E
hBMMSCs
BSO(mM)
F
TGFβ1
R²=0.5306
ng/mL
Age
G
TGFβ2
R²=0.2791
H
TGFβ3
R²=0.5897
I
TGFβ1
pg/mL
Month
J
TGFβ2
K
TGFβ3
L
BMMSCs
TGFβ1(ng/ml)
M
hBMMSCs
TGFβ1(ng/ml)
N
Angptl8
mRNA expression (relative to CON)
TGFβ1
siSmad3

Supplemental Figure 2. ANGPTL8 expression increased with age in bone marrow during skeletal aging.

(A) Representative dot plot of BMMSCs FACS employing expression (+) or absence (-) of standard markers: CD11b-CD45-CD29+Sca1+. (B-C) qPCR analysis of the levels of p16 and *Angptl8* expression in mice BMMSCs with the administration of 17β-estradiol and BSO. (D-E) qPCR analysis of the levels of *P16* and *ANGPTL8* expression in human BMMSCs with the administration of 17β-estradiol and BSO. (F-H) TGFβ1, 2 and 3 levels in human bone marrow samples were detected by ELISA. (I-K) TGFβ1, 2 and 3 levels in serum from 3, 12 and 24-month-old wild-type mice were detected by ELISA. (L) qPCR analysis of the levels of *p16* and *Angptl8* expression in mice BMMSCs with the administration of TGF-β1. (M) qPCR analysis of the levels of *P16* and *ANGPTL8* expression in human BMMSCs with the administration of TGF-β1. (N) qRT-PCR of *Angptl8* relative expression levels from BMMSCs treated with TGFβ1 and siSmad3. n=3. Data are mean ± SEM.*P<0.05; **P<0.01; ***P<0.001;****P<0.0001(one-way ANOVA followed by Tukey's multiple-comparison procedure for multiple group comparison).

A

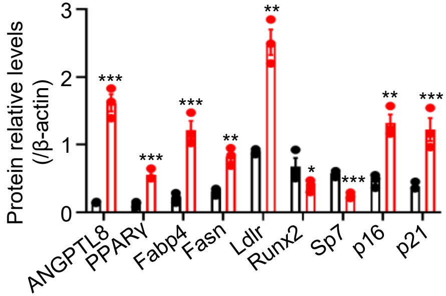

Supplemental Figure 3. ANGPTL8 modulated cell-fate choice of MSCs between adipocytes and osteoblasts.
(A) ANGPTL8, PPARγ, Fabp4, Fasn, Ldlr, Runx2, Sp7, p16 and p21 expression relative to β-actin were assessed by densitometric analysis from BMMSCs transfected with lentiviral LV-GFP or LV-ANGPTL8. n=3. Data are mean ± SEM. *:P<0.05; **:P<0.01; ***:P<0.001; ****:P<0.0001. (Student's t test).

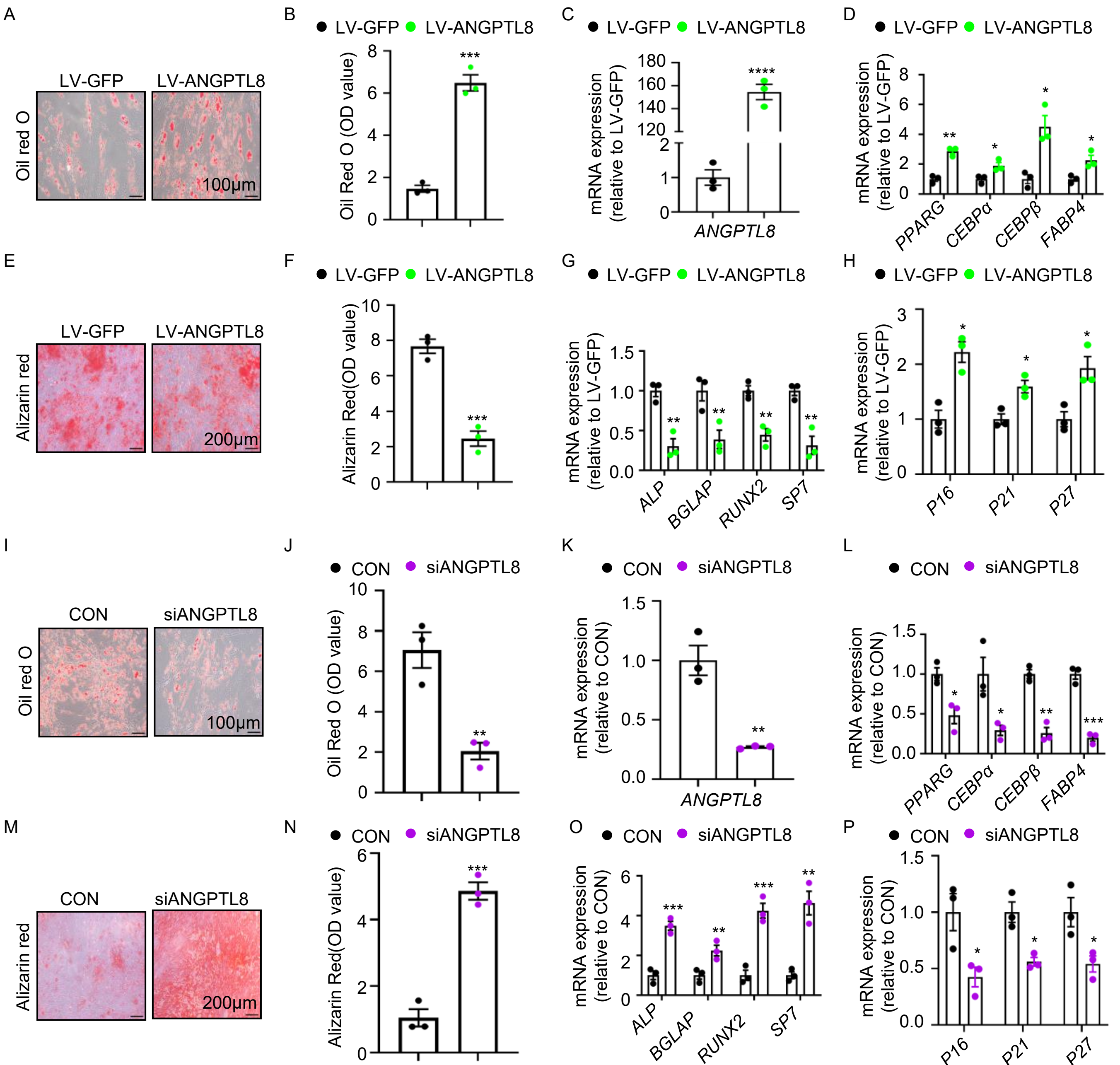
A
LV-GFP
LV-ANGPTL8
Oil red O
100μm
B
LV-GFP
LV-ANGPTL8
Oil Red O (OD value)
***
C
mRNA expression (relative to LV-GFP)
****
ANGPTL8
D
mRNA expression (relative to LV-GFP)
**
*
*
*
PPARG
CEBPα
CEBPβ
FABP4
E
Alizarin red
200μm
F
Alizarin Red(OD value)
***
G
**
ALP
BGLAP
RUNX2
SP7
H
P16
P21
P27
I
CON
siANGPTL8
Oil red O
100μm
J
Oil Red O (OD value)
**
K
mRNA expression (relative to CON)
ANGPTL8
L
***
M
Alizarin red
200μm
N
Alizarin Red(OD value)
O
P

Supplemental Figure 4. ANGPTL8 modulated cell-fate choice of hBMMSCs between adipocytes and osteoblasts.

(A) Cell differentiation was assessed 14 days after adipogenic induction by oil red O staining (scale bar: 100μm).

(B) Quantification of oil red O based on oil red O staining (A).

(C) qRT-PCR analysis of the mRNA expression of *ANGPTL8* in hBMMSCs after transfection with lentiviral LV-GFP or LV-ANGPTL8.

(D) qRT-PCR analysis of the mRNA expression of adipogenic markers (*PPARG, CEBPα, CEBPβ* and *FABP4*) in ANGPTL8-overexpressing hBMMSCs.

(E) Cell differentiation was assessed 14 days after osteogenic induction by alizarin red staining (scale bar: 200μm).

(F) Quantification of calcium mineralization based on alizarin red staining (E).

(G) qRT-PCR analysis of the mRNA expression of osteogenic markers (*ALP, BGLAP, RUNX2* and *SP7*) in ANGPTL8-overexpressing hBMMSCs.

(H) qRT-PCR analysis of the mRNA of the aging markers, *P16, P21* and *P27* in ANGPTL8-overexpressing hBMMSCs.

(I) The adipogenic of hBMMSCs after transfected with siANGPTL8 was assessed 14 days after induction of differentiation by oil red O (scale bar: 100μm).

(J) Quantification of oil red O based on oil red O staining (I).

(K) qRT-PCR analysis of the mRNA expression of *ANGPTL8* in hBMMSCs after transfection with siANGPTL8.

(L) Expression of adipogenic (*PPARG, CEBPα, CEBPβ* and *FABP4*) as assessed by qRT-PCR of induced hBMMSCs.

(M) The osteogenic potential of hBMMSCs after transfected with siANGPTL8 was assessed 14 days after induction of differentiation by alizarin red staining (scale bar: 200μm).

(N) Quantification of calcium mineralization based on alizarin red staining (M).

(O) Expression osteogenic (*ALP, BGLAP, RUNX2,* and *SP7*) markers as assessed by qRT-PCR of induced hBMMSCs.

(P) qRT-PCR analysis of the mRNA expression of the senescence markers, *P16, P21* and *P27* in human BMMSCs after transfection with siANGPTL8.

n = 3 biologically independent hBMMSCs samples. Data are mean ± SEM. *:P<0.05; **:P<0.01; ***:P<0.001; ****:P<0.0001. (Student's t test).

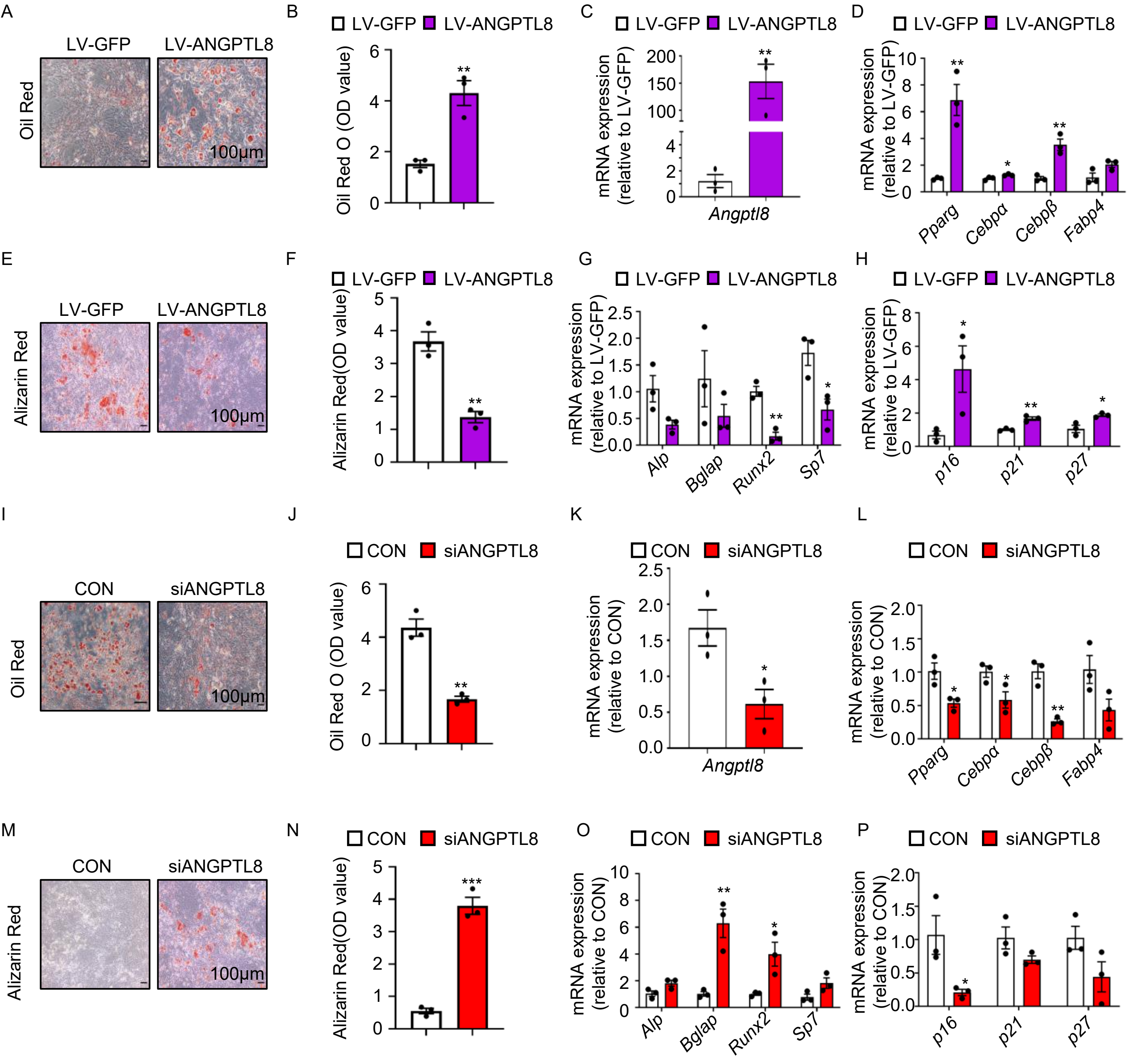
A
LV-GFP
LV-ANGPTL8
Oil Red
100μm
B
LV-GFP
LV-ANGPTL8
Oil Red O (OD value)
C
mRNA expression
(relative to LV-GFP)
Angptl8
D
Pparg
Cebpα
Cebpβ
Fabp4
E
Alizarin Red
F
Alizarin Red(OD value)
G
Alp
Bglap
Runx2
Sp7
H
p16
p21
p27
I
CON
siANGPTL8
J
K
mRNA expression
(relative to CON)
L
M
N
O
P

Supplemental Figure 5. ANGPTL8 modulated cell-fate choice of C3H10T1/2 cells between adipocytes and osteoblasts.
(A) Cell differentiation was assessed 14 days after adipogenic induction by oil red O staining (scale bar: 100μm).
(B) Quantification of oil red O based on oil red O staining (A).
(C) qRT-PCR analysis of the mRNA expression of *Angptl8* in C3H10T1/2 cells after transfection with lentiviral LV-GFP or LV-ANGPTL8.
(D) qRT-PCR analysis of the mRNA expression of adipogenic markers (*Pparg, Cebpα, Cebpβ* and *Fabp4*) in ANGPTL8-overexpressing C3H10T1/2 cells.
(E) Cell differentiation was assessed 14 days after osteogenic induction by alizarin red staining (scale bar: 100μm).
(F) Quantification of calcium mineralization based on alizarin red staining (E).
(G) qRT-PCR analysis of the mRNA expression of osteogenic markers (*Alp, Bglap, Runx2* and *Sp7*) in ANGPTL8-overexpressing C3H10T1/2 cells.
(H) qRT-PCR analysis of the mRNA of the aging markers, *p16, p21* and *p27* in ANGPTL8-overexpressing C3H10T1/2 cells.
(I) The adipogenic of C3H10T1/2 cells after transfected with siANGPTL8 was assessed 14 days after induction of differentiation by oil red O (scale bar: 100μm).
(J) Quantification of oil red O based on oil red O staining (I).
(K) qRT-PCR analysis of the mRNA expression of *Angptl8* in C3H10T1/2 cells after transfection with siANGPTL8.
(L) Expression of adipogenic (*Pparg, Cebpα, Cebpβ* and *Fabp4*) as assessed by qRT-PCR of induced C3H10T1/2 cells.
(M) The osteogenic potential of C3H10T1/2 cells after transfected with siANGPTL8 was assessed 14 days after induction of differentiation by alizarin red staining (scale bar: 100μm).
(N) Quantification of calcium mineralization based on alizarin red staining (M).
(O) Expression osteogenic (*Alp, Bglap, Runx2* and *Sp7*) markers as assessed by qRT-PCR of induced C3H10T1/2 cells.
(P) qRT-PCR analysis of the mRNA expression of the senescence markers, *p16, p21* and *p27* in C3H10T1/2 cells after transfection with siANGPTL8.
n = 3 biologically independent C3H10T1/2 cells samples. Data are mean ± SEM. *:$P<0.05$; **:$P<0.01$; ***:$P<0.001$; ****:$P<0.0001$. (Student's t test).

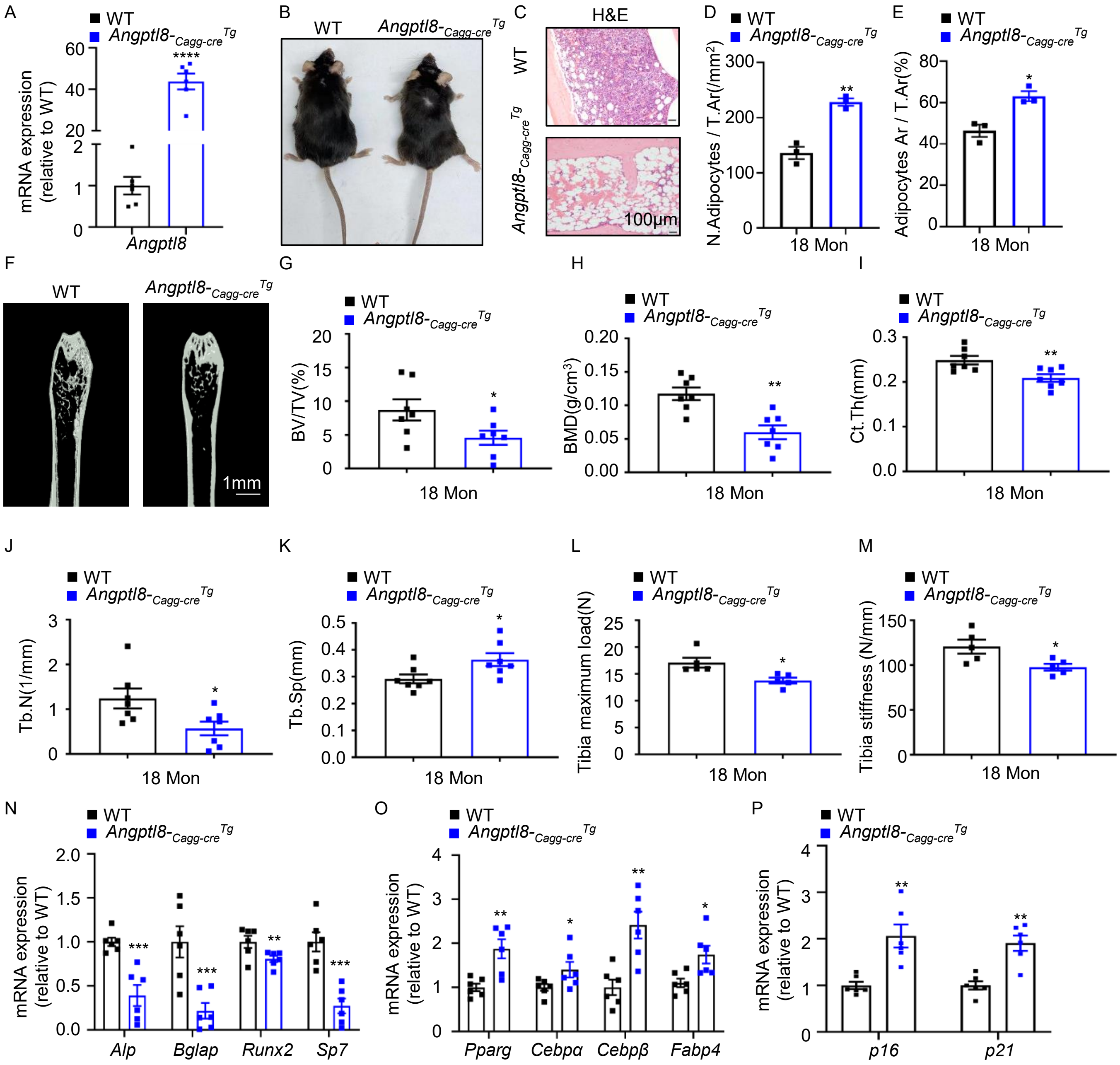

A
WT
Angptl8-Cagg-cre Tg
mRNA expression (relative to WT)
****
Angptl8
B
WT
Angptl8-Cagg-cre Tg
C
H&E
WT
Angptl8-Cagg-cre Tg
100μm
D
N.Adipocytes / T.Ar(/mm2)
**
18 Mon
E
Adipocytes Ar / T.Ar(%)
*
18 Mon
F
WT
Angptl8-Cagg-cre Tg
1mm
G
BV/TV(%)
18 Mon
H
BMD(g/cm3)
18 Mon
I
Ct.Th(mm)
18 Mon
J
Tb.N(1/mm)
18 Mon
K
Tb.Sp(mm)
18 Mon
L
Tibia maximum load(N)
18 Mon
M
Tibia stiffness (N/mm)
18 Mon
N
mRNA expression (relative to WT)
Alp
Bglap
Runx2
Sp7
O
mRNA expression (relative to WT)
Pparg
Cebpα
Cebpβ
Fabp4
P
mRNA expression (relative to WT)
p16
p21

Supplemental Figure 6. *Angptl8-$_{Cagg\text{-}cre}$^{Tg}* mice exhibited lower bone mass and higher bone marrow fat accumulation.

(A) qRT-PCR mRNA analysis of the mRNA expression of *Angptl8* in bone marrow of WT and *Angptl8-$_{Cagg\text{-}cre}$^{Tg}* male mice.

(B) Representative images of WT and *Angptl8-$_{Cagg\text{-}cre}$^{Tg}* male mice at 18-month-old.

(C) H&E staining for bone marrow tissue in the femurs of WT and *Angptl8-$_{Cagg\text{-}cre}$^{Tg}* male mice (scale bar:100μm).

(D-E) Quantification of number (D) and area of adipocytes (E) in femurs (n = 5).

(F) Representative microCT images of femurs from WT and *Angptl8-$_{Cagg\text{-}cre}$^{Tg}* male mice (Scale bar：1mm).

(G-K) Quantitative microCT analysis of femurs from WT and *Angptl8-$_{Cagg\text{-}cre}$^{Tg}* male mice (BV/TV, bone volume per tissue volume; Ct.Th, cortical bone thickness, BMD, bone mineral density, Tb.N, trabecular number; Tb.Sp, trabecular separation). n = 6.

(L-M) Three-point bending measurement of tibia maximum load (K) and stiffness (L). n = 5. (N-P) qRT-PCR mRNA analysis of the mRNA expression of osteogenic (*Alp, Bglap, Runx2* and *Sp7*), adipogenic (*Pparg, Cebpα, Cebpβ* and *Fabp4*) and aging (*p16, p21*) markers WT and *Angptl8-$_{Cagg\text{-}cre}$^{Tg}* male mice. n = 6. Data are mean ± SEM. *:P<0.05; **:P<0.01;***:P<0.001;****:P<0.0001. (Student's t test).

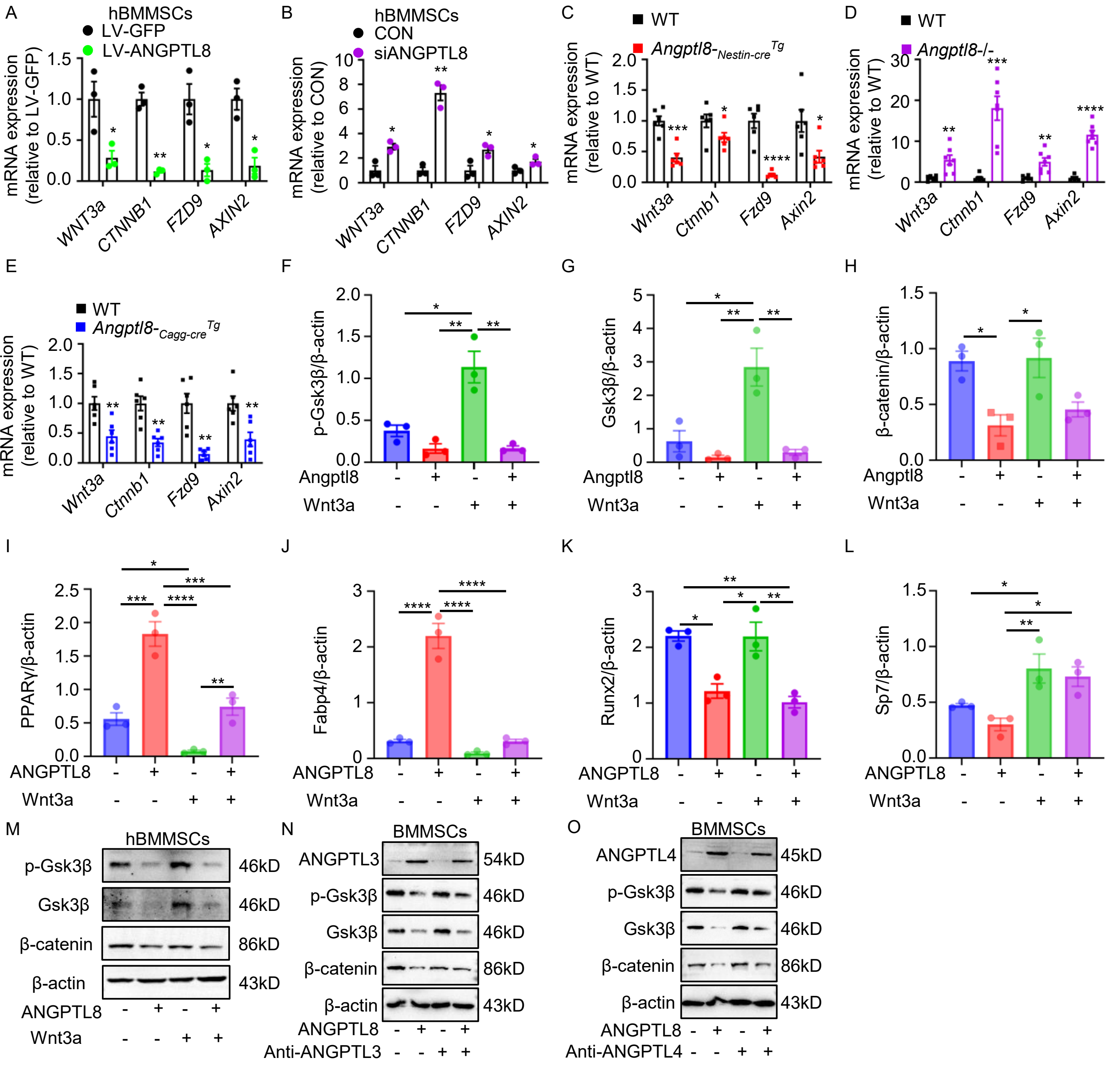

A
hBMMSCs
LV-GFP
LV-ANGPTL8
mRNA expression (relative to LV-GFP)
WNT3a
CTNNB1
FZD9
AXIN2
B
hBMMSCs
CON
siANGPTL8
mRNA expression (relative to CON)
C
WT
Angptl8-Nestin-cre Tg
mRNA expression (relative to WT)
Wnt3a
Ctnnb1
Fzd9
Axin2
D
WT
Angptl8-/-
E
WT
Angptl8-Cagg-cre Tg
F
p-Gsk3β/β-actin
Angptl8
Wnt3a
G
Gsk3β/β-actin
H
β-catenin/β-actin
I
PPARγ/β-actin
ANGPTL8
J
Fabp4/β-actin
K
Runx2/β-actin
L
Sp7/β-actin
M
hBMMSCs
p-Gsk3β 46kD
Gsk3β 46kD
β-catenin 86kD
β-actin 43kD
N
BMMSCs
ANGPTL3 54kD
Anti-ANGPTL3
O
ANGPTL4 45kD
Anti-ANGPTL4

Supplemental Figure 7. ANGPTL8 regulated MSCs differentiation by Wnt/β-catenin signaling pathway.

(A) qRT-PCR analysis of the mRNA expression *of WNT3a, FZD9, AXIN2* and *CTNNB1* after hBMMSCs transfection with LV-GFP and LV-ANGPTL8. n = 3.

(B) qRT-PCR analysis of the mRNA expression of *WNT3a, FZD9, AXIN2* and *CTNNB1* after hBMMSCs transfection with siANGPTL8. n = 3.

(C) qRT-PCR analysis of the mRNA expression of *Wnt3a, Fzd9, Axin2* and *Ctnnb1* from WT and *Angptl8-*$_{Nestin\text{-}cre}{}^{Tg}$ male mice. n = 6.

(D) qRT-PCR analysis of the mRNA expression of *Wnt3a, Fzd9, Axin2* and *Ctnnb1* from WT and *Angptl8-/-* male mice. n = 6.

(E) qRT-PCR analysis of the mRNA expression of *Wnt3a, Fzd9, Axin2* and *Ctnnb1* from WT and *Angptl8-*$_{Cagg\text{-}cre}{}^{Tg}$ male mice. n = 6.

(F-L) p-Gsk3β, Gsk3β, β-catenin, PPARγ、Fabp4、Runx2 and Sp7 expression relative to *β*-actin were assessed by densitometric analysis from BMMSCs treated with ANGPTL8 and Wnt3a. N = 3.

(M) ANGPTL8 blunted Wnt3a-induced phosphorylation of Gsk3β and prevented the subsequent accumulation of β-catenin in hBMMSCs. n = 3.

(N) Western blotting of ANGPTL3, p-Gsk3β, Gsk3β, and β-catenin from BMMSCs treated with ANGPTL8 and Anti-ANGPTL3.

(O) Western blotting of ANGPTL4, p-Gsk3β, Gsk3β, and β-catenin from BMMSCs treated with ANGPTL8 and Anti-ANGPTL4.

Data are mean ± SEM. *:P<0.05; **:P<0.01;***:P<0.001;****:P<0.0001(two-way ANOVA followed by Tukey's multiple-comparison procedure for multiple group comparison and two-tailed Student’s t test for 2 group comparison).

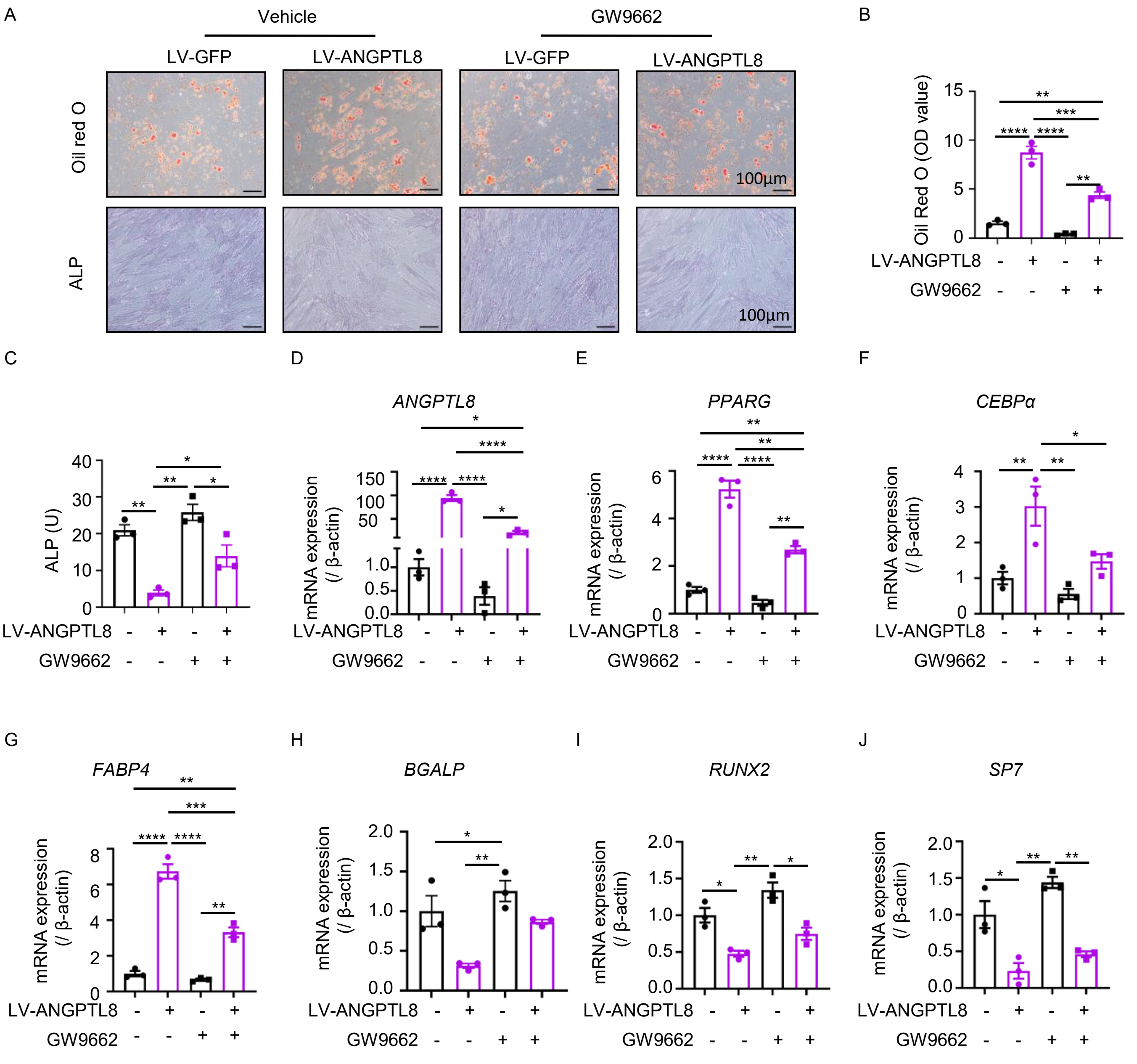
A
Vehicle
GW9662
LV-GFP
LV-ANGPTL8
LV-GFP
LV-ANGPTL8
Oil red O
ALP
100µm
100µm
B
Oil Red O (OD value)
LV-ANGPTL8
GW9662
C
ALP (U)
D
ANGPTL8
mRNA expression (/ β-actin)
E
PPARG
F
CEBPα
G
FABP4
H
BGALP
I
RUNX2
J
SP7

Supplemental Figure 8. PPARγ inhibition partially inhibits ANGPTL8 expression and rescues the phenotype of ANGPTL8-overexpression in hBMMSCs.

(A) Representative images of oil red O staining (Scale bar:100μm) and ALP staining (Scale bar:100μm) in hBMMSCs after transfected with LV-GFP and LV-ANGPTL8 treated with vehicle or a PPARγ inhibitor (GW9662).

(B) Quantification of oil red O based on oil red O staining (A).

(C) Quantification of alkaline phosphatase based on ALP staining (A).

(D) qRT-PCR analysis of the mRNA expression of *ANGPTL8* in treated hBMMSCs.

(E-G) qRT-PCR analyses of the mRNA expression *of PPARG, CEBPα,* and *Fabp4* in treated hBMMSCs under adipogenic conditions.

(H-J) qRT-PCR analysis of the mRNA expression of *BGALP, RUNX2*, and *SP7* in treated hBMMSCs under osteogenic conditions.

n = 3 biologically independent hBMMSCs samples. Data are mean ± SEM. *:P<0.05; **:P<0.01;***:P<0.001;****:P<0.0001(two-way ANOVA followed by Tukey's multiple-comparison procedure for multiple group comparison).

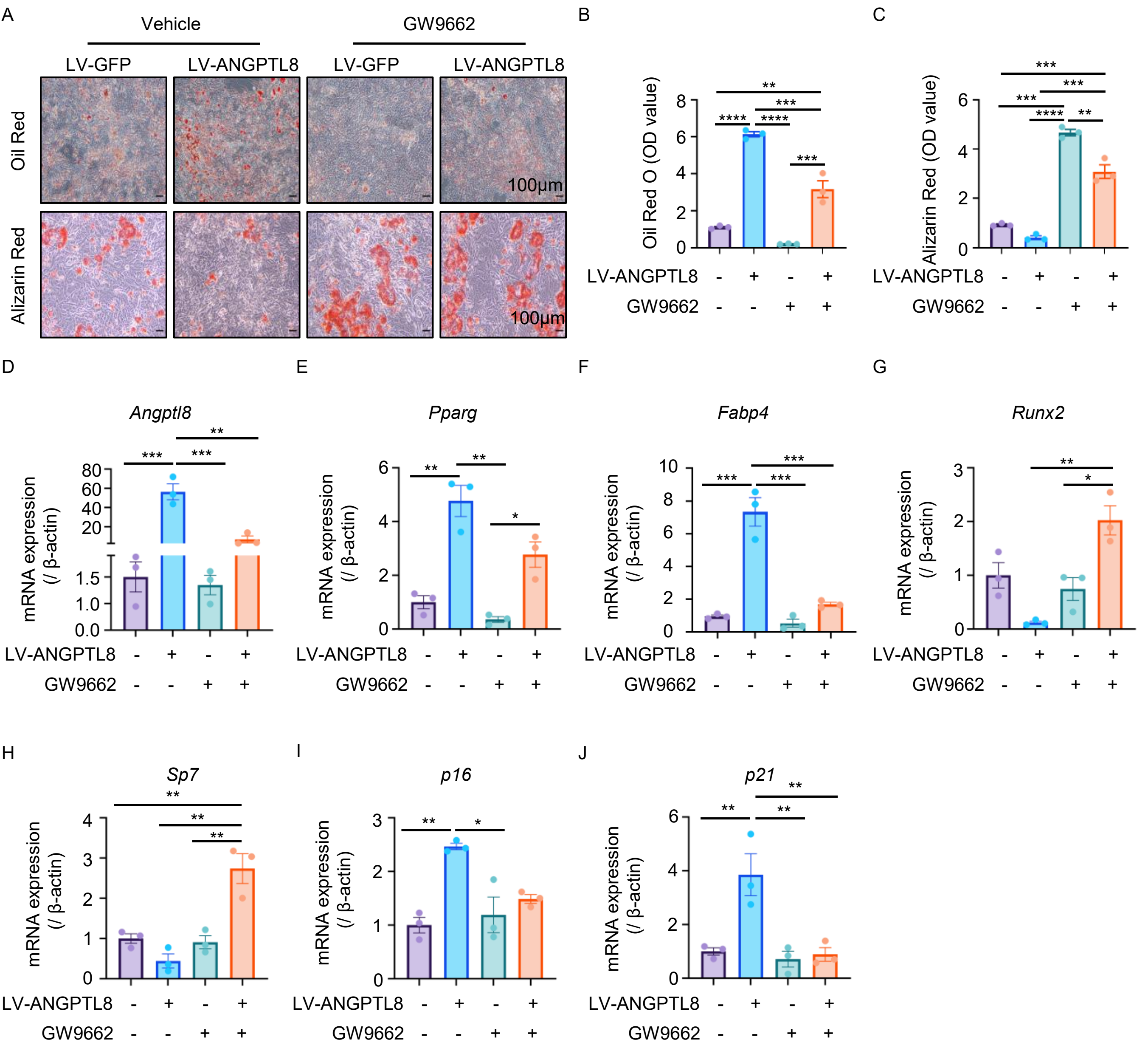
A
Vehicle
GW9662
LV-GFP
LV-ANGPTL8
Oil Red
Alizarin Red
100μm
B
Oil Red O (OD value)
C
Alizarin Red (OD value)
LV-ANGPTL8
GW9662
D
Angptl8
E
Pparg
F
Fabp4
G
Runx2
mRNA expression (/ β-actin)
H
Sp7
I
p16
J
p21

Supplemental Figure 9. PPARγ inhibition partially inhibits ANGPTL8 expression and rescues the phenotype of ANGPTL8-overexpression in C3H10T1/2 cells.

(A) Representative images of oil red O staining (Scale bar:100μm) and alizarin red staining (Scale bar:100μm) in C3H10T1/2 cells after transfected with LV-GFP and LV-ANGPTL8 treated with vehicle or a PPARγ inhibitor (GW9662).

(B) Quantification of oil red O based on oil red O staining (A).

(C) Quantification of calcium mineralization based on alizarin red staining (A).

(D) qRT-PCR analysis of the mRNA expression of *Angptl8* in treated C3H10T1/2 cells.

(E-F) qRT-PCR analyses of the mRNA expression *of Pparg* and *Fabp4* in treated C3H10T1/2 cells under adipogenic conditions.

(G-H) qRT-PCR analysis of the mRNA expression of *Runx2*, and *Sp7* in treated C3H10T1/2 cells under osteogenic conditions.

(I-J) qRT-PCR analysis of the mRNA expression *of p16* and *p21* in treated C3H10T1/2 cells. n = 3 biologically independent C3H10T1/2 cells.

Data are mean ± SEM. *:P<0.05; **:P<0.01;***:P<0.001;****:P<0.0001(two-way ANOVA followed by Tukey's multiple-comparison procedure for multiple group comparison).

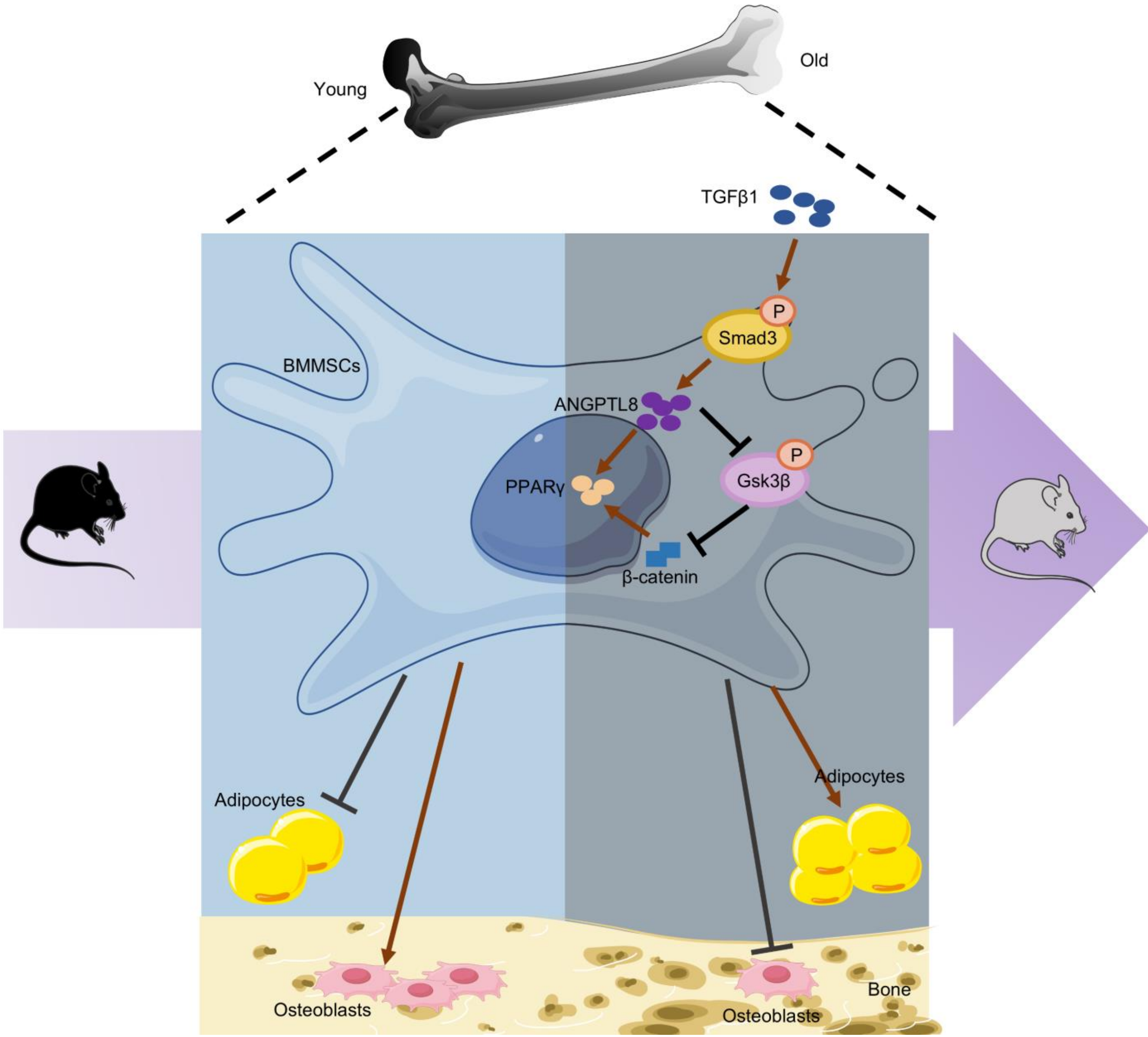
Young
Old
TGFβ1
P
Smad3
BMMSCs
ANGPTL8
P
Gsk3β
PPARγ
β-catenin
Adipocytes
Adipocytes
Osteoblasts
Osteoblasts
Bone

Supplemental Figure 10. During bone aging, TGF-β1 upregulates ANGPTL8 in BMMSCs by phosphorylating its downstream molecule Smad3. Subsequently, ANGPTL8 enhances PPARγ expression while simultaneously inhibiting core molecules of the Wnt/β-catenin signaling pathway—P-Gsk3β, Gsk3β, and β-catenin—thereby exerting regulatory effects on BMMSCs differentiation. Notably, inhibiting the Wnt/β-catenin signaling pathway itself contributes to the upregulation of PPARγ.

Supplementary information of
Angiopoietin-like protein 8 governs osteoblast-adipocyte lineage commitment during skeletal aging

Authors Names: Yaming Guo[1,2,3,5], Zeqing Zhang[1,2,3,5], Junyu He[1,2,3], Peiqiong Luo[1,2,3], Zhihan Wang[1,2,3], Yurong Zhu[1,2,3], Xiaoyu Meng[1,2,3], Limeng Pan[1,2,3], Ranran Kan[1,2,3], Yuxi Xiang[1,2,3], Beibei Mao[1,2,3], Yi He[1,2,3], Siyi Wang[1,2,3], Yan Yang[1,2,3], Fengjing Guo[4], Hongbo You[4], Feng Li[4], Danpei Li[1,2,3*], Yong Chen[1,2,3*], Xuefeng Yu[1,2,3*]

1.Division of Endocrinology, Department of Internal Medicine, Tongji Hospital, Tongji Medical College, Huazhong University of Science and Technology, Wuhan, China.

2.Hubei Clinical Medical Research Center for Endocrinology and Metabolic Diseases, Hubei, China.

3.Branch of National Clinical Research Center for Metabolic Diseases, Hubei, China.

4.Department of Orthopedics, Tongji Hospital, Tongji Medical College, Huazhong University of Science and Technology, Wuhan 430030, China.

5.Contribution equally.

[*]**Corresponding author:**

Danpei Li, M.D., Ph.D., Division of Endocrinology, Department of Internal Medicine, Tongji Hospital, Tongji Medical College, Huazhong University of Science and Technology, 1095 Jiefang Avenue, Wuhan 430030, China.

Email: ldp19940730@163.com.

Yong Chen, M.D., Ph.D., Professor of Medicine, Division of Endocrinology, Department of Internal Medicine, Tongji Hospital, Tongji Medical College, Huazhong University of Science and Technology, 1095 Jiefang Avenue, Wuhan 430030, China.

Email: tj.y.chen@vip.163.com.

Xuefeng Yu, M.D., Ph.D., Professor of Medicine, Division of Endocrinology, Department of Internal Medicine, Tongji Hospital, Tongji Medical College, Huazhong University of Science and Technology, 1095 Jiefang Avenue, Wuhan 430030, China.

Email: xfyu188@163.com.

Table 1. Primers for quantitative RT-PCR

Species	Name	Sequence
Mouse	*Angptl8-F*	CTGACCCTGCTCTTTCACGG
	Angptl8-R	GCTCTGTCATAGAGGCCCAG
	β-actin-F	GGCTGTATTCCCCTCCATCG
	β-actin-R	CCAGTTGGTAACAATGCCATGT
	P16-F	CTAGAGAGGATCTTGAGAAGAGGGC
	P16-R	TAGTTGAGCAGAAGAGCTGCTACGT
	P21-F	GAACATCTCAGGGCCGAAAAC
	P21-R	CTGCGCTTGGAGTGATAGAA
	P27-F	TCTCTTCGGCCCGGTCAAT
	P27-R	AAATTCCACTTGCGCTGACTC
	P53-F	CTCTCCCCCGCAAAAGAAAAA
	P53-R	CGGAACATCTCGAAGCGTTTA
	Pparg-F	GGAAAGACAACGGACAAATCAC
	Pparg-R	TACGGATCGAAACTGGCAC
	Cebpα-F	TGGACAAGAACAGCAACGAG
	Cebpα-R	TCACTGGTCAACTCCAGCAC
	Cebpβ-F	CGACTTCAGCGCCTACATTGA
	Cebpβ-R	GAAGAGGTCGGCGAAGAGTT
	Fabp4-F	AAGGTGAAGAGCATCATAACCCT

	Fabp4-R	TCACGCCTTTCATAACACATTCC
	Alp-F	GGCTGGAGATGGACAAATTCC
	Alp-R	CCGAGTGGTAGTCACAATGCC
	Bglap-F	TTGGTGCACACCTAGCAGAC
	Bglap-R	ACCTTATTGCCCTCCTGCTT
	Runx2-F	CCGGGAATGATGAGAACTA
	Runx2-R	ACCGTCCACTGTCACTTT
	Sp7-F	CTCTCTGCTTGAGGAAGAAG
	Sp7-R	GTCCATTGGTGCTTGAGAAG
	Wnt3a-F	CAGGAACTACGTGGAGATCATGC
	Wnt3a-R	CGTGTCACTGCGAAAGCTACT
	Ctnnb-F	CCCAGTCCTTCACGCAAGAG
	Ctnnb-R	CATCTAGCGTCTCAGGGAACA
	Axin2-F	AACCTATGCCCGTTTCCTCTA
	Axin2-R	GAGTGTAAAGACTTGGTCCACC
	Fzd9-F	TTGCTCTATTATTTCGGGATGGC
	Fzd9-R	CAGGACCACGATAGTTTTGAGTG
	Fasn-F	TGGGTAATCCATAGAGCCCAG
	Fasn-R	TTCTTGCGATACACTCTGGTGC
	Ldlr-F	GAAGGCAGCTACAAGTGTGAG
	Ldlr-R	GGGGAGCAGACTGGTGTACT
Human	*ANGPTL8-F*	AGAAGGTGCTACGGGACAG

	ANGptL8-R	AGCGTGAGCCTTTAAGACCTC
	β-ACTIN-F	CATGTACGTTGCTATCCAGGC
	β-ACTIN-R	CTCCTTAATGTCACGCACGAT
	P16-F	GATCCAGGTGGGTAGAAGGTC
	P16-R	CCCCTGCAAACTTCGTCCT
	P21-F	TGTCCGTCAGAACCCATGC
	P21-R	AAAGTCGAAGTTCCATCGCTC
	P27-F	TAATTGGGGCTCCGGCTAACT
	P27-R	TGCAGGTCGCTTCCTTATTCC
	P53-F	GAGGTTGGCTCTGACTGTACC
	P53-R	TCCGTCCCAGTAGATTACCAC
	PPARG-F	ACCAAAGTGCAATCAAAGTGGA
	PPARG-R	ATGAGGGAGTTGGAAGGCTCT
	CEBPα-F	GCGGGAACGCAACAACATC
	CEBPα-R	GTCACTGGTCAACTCCAGCAC
	CEBPβ-F	CTTCAGCCCGTACCTGGAG
	CEBPβ-R	GGAGAGGAAGTCGTGGTGC
	FABP4-F	ACTGGGCCAGGAATTTGACG
	FABP4-R	CTCGTGGAAGTGACGCCTT
	ALP-F	ACCACCACGAGAGTGAACCA
	ALP-R	CGTTGTCTGAGTACCAGTCCC
	BGLAP-F	CACTCCTCGCCCTATTGGC

	BGLAP-R	CCCTCCTGCTTGGACACAAAG
	RUNX2-F	TGGTTACTGTCATGGCGGGTA
	RUNX2-R	TCTCAGATCGTTGAACCTTGCTA
	SP7-F	AACCCCCAGCTGCCCACCTACC
	SP7-R	GACGCTCCAGCTCATCCGAACG
	WNT3a-F	AGCTACCCGATCTGGTGGTC
	WNT3a-R	CAAACTCGATGTCCTCGCTAC
	CTNNB-F	AGCTTCCAGACACGCTATCAT
	CTNNB-R	CGGTACAACGAGCTGTTTCTAC
	AXIN2-F	TACACTCCTTATTGGGCGATCA
	AXIN2-R	TTGGCTACTCGTAAAGTTTTGGT
	FZD9-F	TGCGAGAACCCCGAGAAGT
	FZD9-R	GGGACCAGAACACCTCGAC
