# Supplementary material for: Angiopoietin-like 8 governs osteoblast-adipocyte lineage commitment during skeletal aging
Source: JCI Insight. 2025 Oct 21;10(23):e189371. doi: 10.1172/jci.insight.189371 (PMC12890479; doi:10.1172/jci.insight.189371)

Figure 1I

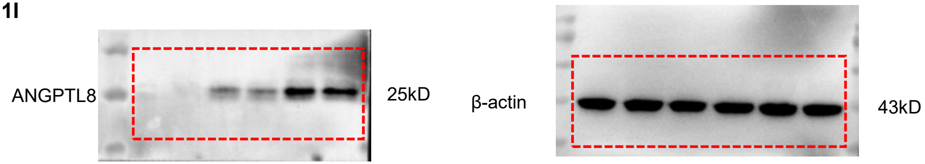

Figure 2F

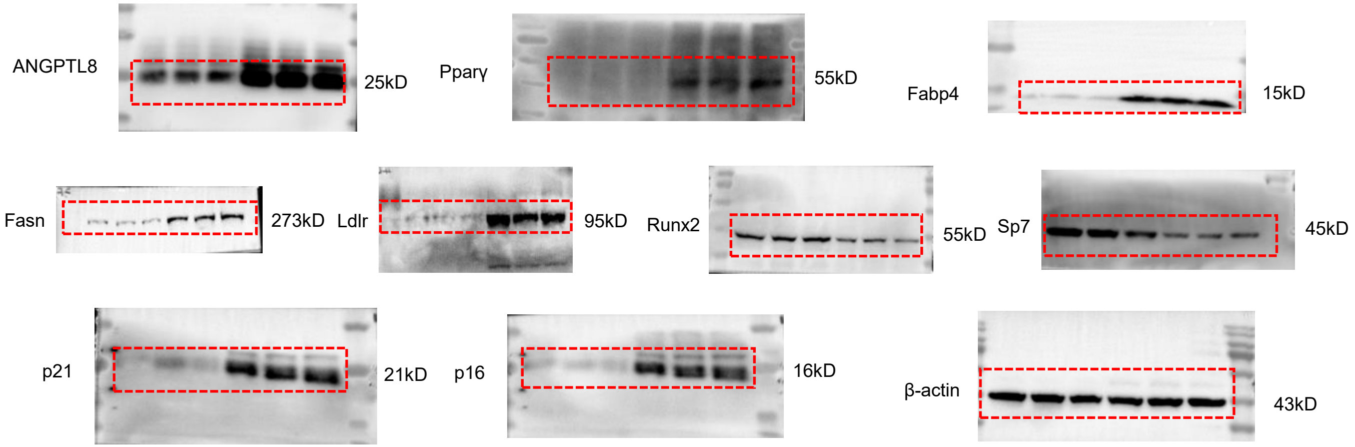

Figure 2M

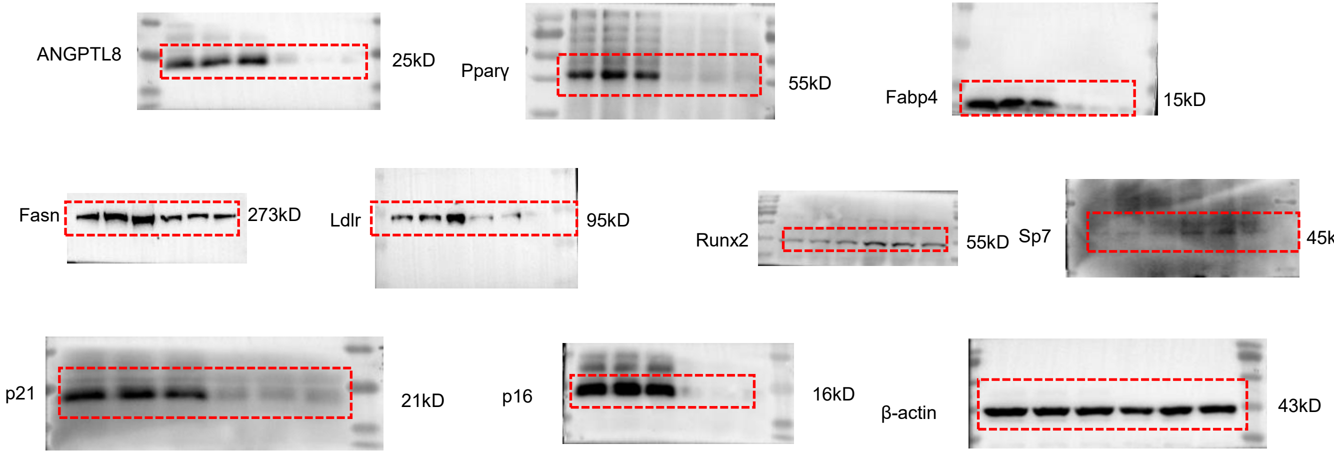

Figure 5D

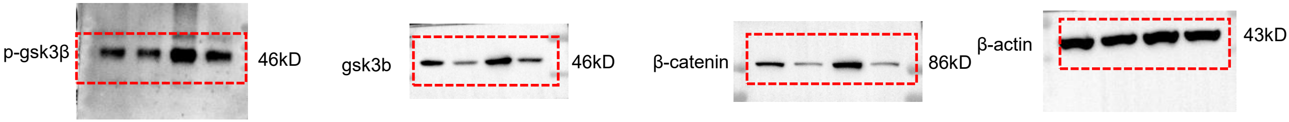

Figure 5E

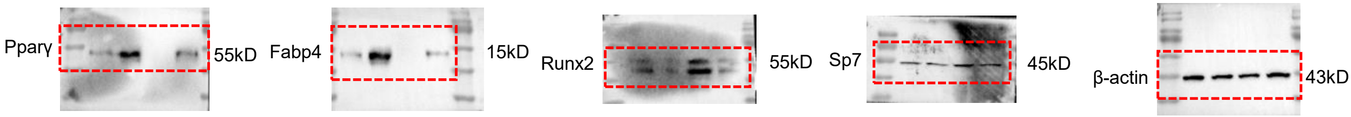

Figure 5J

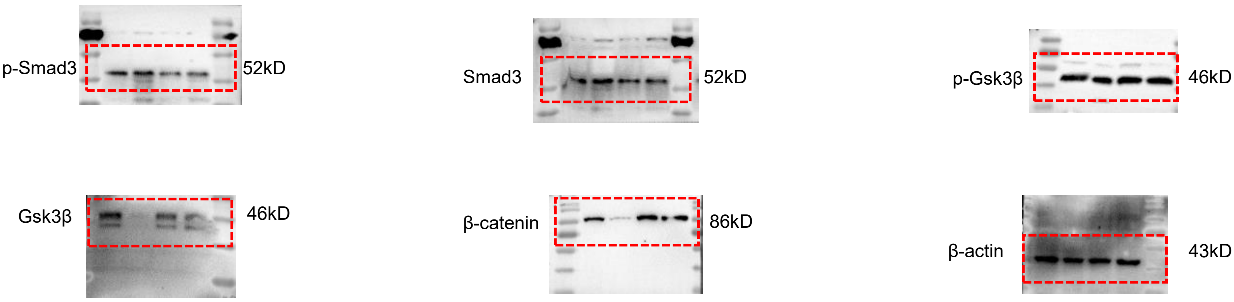

Supplemental Figure 7M

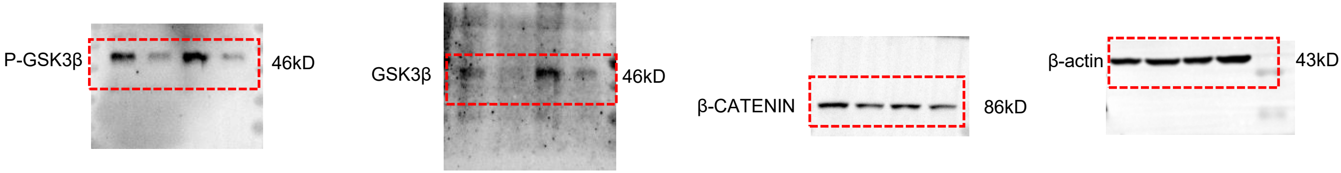

Supplemental Figure 7N

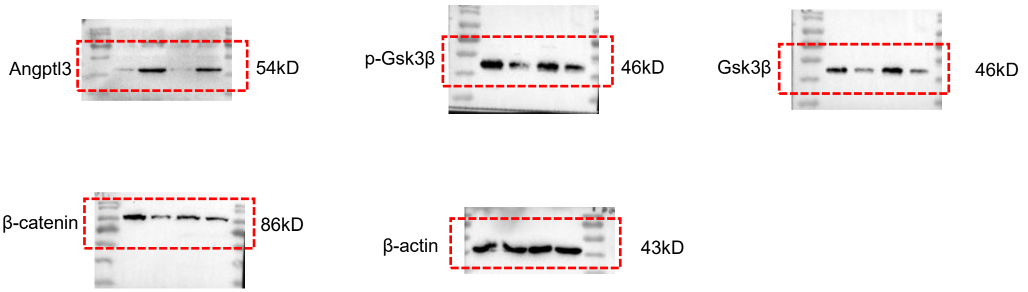

Supplemental Figure 7O

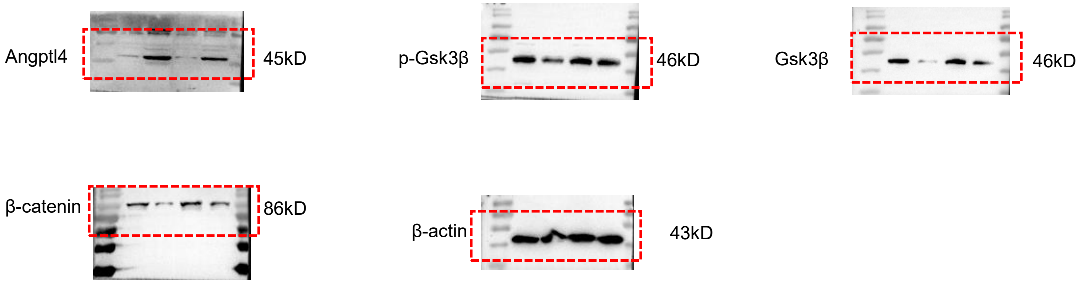

Supplement: Unedited blot and gel images [file jciinsight-10-189371-s140.pdf]
